# The Latent Structure of Interpersonal Problems: Validity of Dimensional, Categorical, and Hybrid Models

**DOI:** 10.1037/abn0000460

**Published:** 2019-09-26

**Authors:** Leon P. Wendt, Aidan G. C. Wright, Paul A. Pilkonis, Tobias Nolte, Peter Fonagy, P. Read Montague, Cord Benecke, Tobias Krieger, Johannes Zimmermann

**Affiliations:** 1 Psychologische Hochschule Berlin; 2 Department of Psychology, University of Pittsburgh; 3 Department of Psychiatry, University of Pittsburgh School of Medicine; 4 Anna Freud National Centre for Children and Families, London, and Research Department of Clinical, Educational and Health Psychology, and the Wellcome Centre for Human Neuroimaging, Institute for Neurology, University College London; 5 Research Department of Clinical, Educational, and Health Psychology, University College London, and Fralin Biomedical Research Institute, Virginia Tech; 6 Department of Psychology, University of Kassel; 7 Department of Psychology, University of Bern; 8 Psychologische Hochschule Berlin

**Keywords:** interpersonal problems, factor mixture modeling, nonnormal factor distribution, confirmatory factor analysis, latent class analysis

## Abstract

Interpersonal problems are key transdiagnostic constructs in psychopathology. In the past, investigators have neglected the importance of operationalizing interpersonal problems according to their latent structure by using divergent representations of the construct: (a) computing scores for severity, agency, and communion (“dimensional approach”), (b) classifying persons into subgroups with respect to their interpersonal profile (“categorical approach”). This hinders cumulative research on interpersonal problems, because findings cannot be integrated both from a conceptual and a statistical point of view. We provide a comprehensive evaluation of interpersonal problems by enlisting several large samples (*N*s = 5,400, 491, 656, and 712) to estimate a set of latent variable candidate models, covering the spectrum of purely dimensional (i.e., confirmatory factor analysis using Gaussian and nonnormal latent *t*-distributions), hybrid (i.e., semiparametric factor analysis), and purely categorical approaches (latent class analysis). Statistical models were compared with regard to their structural validity, as evaluated by model fit (corrected Akaike’s information criterion and the Bayesian information criterion), and their concurrent validity, as defined by the models’ ability to predict relevant external variables. Across samples, the fully dimensional model performed best in terms of model fit, prediction, robustness, and parsimony. We found scant evidence that categorical and hybrid models provide incremental value for understanding interpersonal problems. Our results indicate that the latent structure of interpersonal problems is best represented by continuous dimensions, especially when one allows for nonnormal latent distributions.

## General Scientific Summary

This study suggests that interpersonal problems are best characterized by continuous differences in severity and interpersonal style (i.e., agency and communion), and not by “types” of people.

Interpersonal problems are key constructs relevant to understanding and characterizing psychopathology that cut across traditional diagnostic categories (American Psychiatric Association, 2013; Hopwood, Wright, Ansell, & Pincus, 2013). For example, interpersonal problems show significant associations with a broad range of psychopathological constructs, such as transdiagnostic dimensions (Girard et al., 2017), maladaptive personality traits (Williams & Simms, 2016; Wright et al., 2012), and core dimensions of personality functioning (Dowgwillo, Roche, & Pincus, 2018; Pincus, 2018). Research on pathoplasticity suggests that mental disorders are embedded in diverse interpersonal dynamics, in which symptoms and interpersonal styles have reciprocal effects on their respective expression and maintenance (Cain, Pincus, & Grosse Holtforth, 2010; Dawood, Dowgwillo, Wu, & Pincus, 2018; Erickson et al., 2016; Przeworski et al., 2011). Treatment research has demonstrated that interpersonal problems can be reduced by psychotherapy (e.g., Horowitz, Rosenberg, & Bartholomew, 1993; McFarquhar, Luyten, & Fonagy, 2018; Quilty, Mainland, McBride, & Bagby, 2013) indicating overall symptomatic change (e.g., Altenstein-Yamanaka, Zimmermann, Krieger, Dörig, & Grosse Holtforth, 2017; Dammann et al., 2016; Huber, Henrich, & Klug, 2007; Renner et al., 2012). Interpersonal problems predict worse treatment outcome (Boswell, Cain, Oswald, McAleavey, & Adelman, 2017; Gomez Penedo, Constantino, Coyne, Bernecker, & Smith-Hansen, 2018; Gomez Penedo, Constantino, Coyne, Westra, & Antony, 2017; McEvoy, Burgess, & Nathan, 2014; Shapiro et al., 1994), possibly explained by weaker therapeutic alliance (Constantino et al., 2010; Zilcha-Mano et al., 2015) and poorer quality of cognitive-emotional processing (Altenstein-Yamanaka et al., 2017). Also, the optimal therapeutic intervention might be dependent on the specific problem content (Gomez Penedo et al., 2017, 2018; Krieg & Tracey, 2016; Newman, Jacobson, Erickson, & Fisher, 2017).

**Figure 1:**
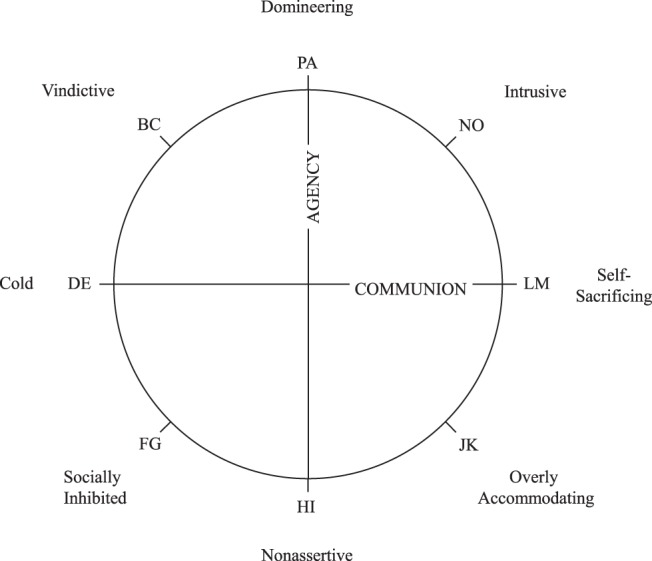
The octant scales of interpersonal problems can be arranged in a circular structural representation, in accordance with the Interpersonal Circumplex (Wiggins, 1991).

The major conceptual foundation of research on interpersonal problems is the Interpersonal Circumplex (IPC; Gurtman & Pincus, 2003; Wiggins, 1991), which delineates interpersonal problems along the dimensions of agency and communion (see Figure 1). However, although this conceptual foundation is well-grounded and established (Acton & Revelle, 2002; Alden, Wiggins, & Pincus, 1990; Boudreaux, Ozer, Oltmanns, & Wright, 2018; Tracey, Rounds, & Gurtman, 1996), investigators adopt distinct scoring procedures across studies, including, but not limited to, dimensional (i.e., computing scores for severity, agency, and communion) and categorical approaches (i.e., classifying persons into subgroups according to their interpersonal profile). In the following, we argue that the way in which researchers conceptualize, operationalize (i.e., score measures), and statistically model interpersonal problems should match their underlying latent structure, namely, it should correspond to what exactly is being measured. Notably, a measurement can be considered valid when the empirical evidence supports that the test scores reflect the target construct adequately (Kane, 2013). Dimensional and categorical scoring procedures of interpersonal problems summarize the information at hand in distinct ways, often based on data reduction techniques, such as factor analysis (i.e., dimensional approach; Altenstein-Yamanaka et al., 2017; Ansell, Grilo, & White, 2012; Barrett & Barber, 2007; Blomquist, Ansell, White, Masheb, & Grilo, 2012; Dinger et al., 2015; Locke et al., 2017; Luo, Nuttall, Locke, & Hopwood, 2018; Miller, Price, Gentile, Lynam, & Campbell, 2012; Puschner, Kraft, & Bauer, 2004; Quilty et al., 2013; Ruiz et al., 2004; Wilson, Revelle, Stroud, & Durbin, 2013) and latent class analysis/cluster analysis (i.e., categorical approach; Cain et al., 2010, 2012; Cooper & Anderson, 2019; Dawood, Thomas, Wright, & Hopwood, 2013; Grosse Holtforth et al., 2014; Hopwood, Clarke, & Perez, 2007; Leihener et al., 2003; Przeworski et al., 2011; Salzer et al., 2008, 2010, 2013; Simon, Cain, Wallner Samstag, Meehan, & Muran, 2015; Wright et al., 2013a; Zilcha-Mano et al., 2015).^1^

Generally, the adequacy of a scoring method depends on whether its implicit structural assumptions are met. Categorical and dimensional approaches to interpersonal problems are mutually exclusive in their structural assumptions and therefore, cannot be equally valid. Failure in meeting those assumptions dilutes the psychometric properties of the measurement and affects the quality of inferences, for instance, by losing statistical power (e.g., median splits) or by researchers being misled to draw false conclusions (Markon, Chmielewski, & Miller, 2011; Morey et al., 2012; Preacher, Rucker, MacCallum, & Nicewander, 2005). To illustrate this, imagine that the latent structure of interpersonal problems was truly three-dimensional, yet a hypothetical finding was based on the categorical approach (i.e., calculating latent classes). In this scenario, the latent classes simply captured blends of the dimensions, in other words, dimensional information on individual differences would have become confounded within classes. How could the statistical association between an interpersonal type and another psychological construct be interpreted under such circumstances? The answer is unclear, because it would be indistinguishable to what degree the association could be attributed to the influence of any one of those three dimensions, let alone additive or interactive effects between them. In addition, empirical results from studies using incompatible operationalizations of the construct cannot be integrated with each other. This is true from a conceptual, but also from a statistical point of view (i.e., meta-analytic integration). As a result of this ambiguity, knowledge on interpersonal problems can hardly be accumulated. The coexistent use of different scoring procedures may amplify information burden and impede comprehension (Kane, 2013). A comprehensive structural analysis of interpersonal problems is needed to resolve those issues and move the field forward—ever more so given the widespread use of the construct as cited above.

**Figure 2 fig2:**
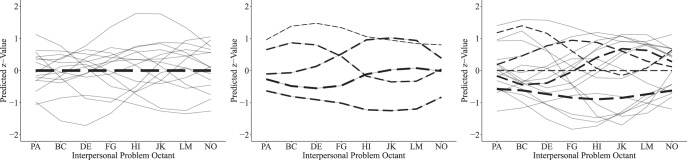
(a)-(c) Predicted octant scores by candidate models (from left to right: CFA-PC, 5-Class LCA, and 3-Factor 5-Class SP-FA) in Sample 1 (*N* = 5,400). Dashed lines indicate the predicted pattern for the average individual in class *k*. Gray lines represent 15 simulated observations. In contrast to our approach to use indicators that were standardized in reference to population norms, the models plotted in this figure were based on *z*-transformed octant scores to facilitate visualization. Line strengths indicate relative class proportions.

Interpersonal problems are commonly measured by the *Inventory of Interpersonal Problems* (IIP; Alden et al., 1990; Horowitz, Rosenberg, Baer, Ureño, & Villaseñor, 1988) and the *Circumplex Scales of Interpersonal Problems* (CSIP; Boudreaux et al., 2018). Both measures assess interpersonal dysfunction on eight octant scales, each denoting a set of social difficulties (see Figure 1). In the following, we will outline the two most commonly used structural models of interpersonal problems. For the fully dimensional approach, a three-factorial solution has been well established (Acton & Revelle, 2002; Alden et al., 1990; Boudreaux et al., 2018; Hopwood & Good, 2018; Monsen, Hagtvet, Havik, & Eilertsen, 2006; Tracey et al., 1996; Wilson et al., 2013). The IPC (Gurtman & Pincus, 2003; Wiggins, 1991) provides the predominant factor rotation, locating the octants within a circular array, as displayed in Figure 1. As a result of the circular representation, the octant scores represent blends of the underlying dimensions. The two orthogonal substantive dimensions of *Agency* and *Communion* form the main axes of this circular arrangement. *Agency* (also: power, control, or dominance) is a bipolar continuum of agentic versus submissive interpersonal tendencies, with high levels indicating an assertive and low levels capturing a nonassertive style. *Communion* (also: solidarity, friendliness, warmth, love, or affiliation) describes a bipolar continuum of communal versus detached interpersonal tendencies, with high levels associated with a warm and low levels associated with a cold style. Some circumplex instruments, such as the IIP and the CSIP, reflect a third dimension that captures the level of severity irrespective of style (Tracey et al., 1996; Wilson et al., 2013). This third dimension denotes a general factor that is reflected by an individual’s mean across all subscales and has been explained to capture overall interpersonal distress (or “elevation” in terms of the Structural Summary Method; Gurtman, 1992; Zimmermann & Wright, 2017). All things combined, the circumplex model predicts a sinusoidal pattern of endorsement on the octants (Figure 2a). Sinusoidal curves are assumed to vary in amplitude, elevation, and (angular) location, depending on the individual’s unobserved continuous factor scores.[Fig-anchor fig2]

The categorical approach to interpersonal problems proposes that the covariance between octants can be sufficiently explained by *k* unobserved interpersonal types (i.e., latent classes). Categorical models estimate a specific pattern of endorsements on the octants for each latent class, respectively. Individuals are assumed to belong to one of the *k* estimated latent classes and are predicted to show the class’ prototypical pattern (i.e., interpersonal prototypes, Figure 2b). Although the categorical approach to interpersonal problems does not presume a circular structural model, the resulting octant profiles are usually summarized and evaluated by circumplex statistics in a subsequent analytic step (i.e., elevation, amplitude, and angular location; Gurtman & Balakrishnan, 1998; Wright, Pincus, Conroy, & Hilsenroth, 2009) to locate latent classes in the circular space (e.g., Cain et al., 2010, 2012; Dawood et al., 2013; Grosse Holtforth et al., 2014; Hopwood et al., 2007; Lo Coco, Gullo, Scrima, & Bruno, 2012; Przeworski et al., 2011; Salzer et al., 2013; Slaney, Pincus, Uliaszek, & Wang, 2006; Wright, Pincus, Conroy, & Elliot, 2009, 2013a; Zilcha-Mano et al., 2015).

Another structural representation of interpersonal problems can be attempted by factor mixture modeling, comprising a spectrum of latent variable models between categorical and dimensional hybrid mixtures (Hallquist & Wright, 2014). Semi-Parametric Factor Analysis (SP-FA) is one such approach, assuming that the covariation between octants can be explained by a mixture of three latent factors and a *k*-fold categorical latent variable. More specifically, SP-FA identifies *k* clusters in which individuals are concentrated in the three-dimensional space. The latent classes can be conceptualized as latent subpopulations that shape a joint multimodal distribution (i.e., *k*-modal).^2^ For example, SP-FA could identify a latent class of individuals characterized by high severity, high agency, and low communion. Hence, the hybrid approach describes individuals both in terms of dimensional scores and class membership (Figure 2c).

We argue that the current practices to operationalize interpersonal problems impede scientific progress for two major reasons: (a) inferences in the research literature may be affected by limited validity and (b) empirical results cannot be integrated conceptually and statistically. Our investigation attempts to resolve those issues by comparing the dimensional, categorical, and hybrid approaches with regard to model fit (structural validity) and prediction (concurrent validity) in four large samples. We aim to facilitate the conceptual and statistical integration of future findings and promote a cumulative science in this important domain of functioning.

## Method

### Samples

An overview of the samples and measures used in this study is displayed in Table 1. Descriptive statistics, measure’s internal consistencies and correlation matrices for each sample are reported in the online supplemental materials (Tables S1–S4).[Table-anchor tbl1]

#### Sample 1

Data from 5,400 treatment-seeking participants (66% women; mean age of 37.40, *SD* = 11.80) were collected at 12 psychoanalytic training institutions belonging to the German Psychoanalytic Society (Benecke et al., 2011; Henkel et al., 2019). Included were participants who underwent assessment and passed entry diagnostics for an outpatient psychotherapy, irrespective of a subsequent beginning of therapy. The mean (standardized) IIP total score was *z* = 0.2.

#### Sample 2

The second sample (Euler et al., 2019) was recruited in Greater London via the Personality and Mood Disorder Research Consortium. The sample (*N* = 491) consisted of 302 healthy community participants and 189 outpatients referred from National Health Service specialist personality disorder clinical services. Participants’ age was *M* = 31.53 (*SD* = 10.74) and 65% were female. We expected a bimodal distribution on psychopathology markers, because outpatients and control group participants were subjected to different sampling processes (i.e., selecting for extreme values). As expected, outpatients were more severely distressed in terms of the IIP total score (*z* = 1.73) than the control group (*z* = 0.41).

#### Sample 3

The full sample consisted of 825 participants from five clinical and community samples collected at the University of Pittsburgh, as described in Girard et al. (2017). Clinical samples were derived from outpatient clinics excluding patients with a lifetime history of psychotic disorders or medical conditions of the central nervous system. One of the samples excluded participants with bipolar disorder. For our analysis, we took a subset of participants that met the criteria for a least one mental disorder diagnosis (*N* = 656). In this sample, the average age was *M* = 35.95 (*SD* = 10.47) and 66% were female. The sample of Axis I + II diagnosed participants was more interpersonally distressed than the national norm, *z* = 0.80 (IIP total). Diagnostic criteria were rated by mental health professionals or trained interviewers on the base of semistructured interviews.

#### Sample 4

We used the second sample from Boudreaux et al. (2018), consisting of 757 undergraduate students (average age was 18.7 years, *SD* = 1.7) that were enrolled at the University of Pittsburgh. Gender was not recorded for most participants because of an administration error (of those who that recorded: 123 women, 77 men). Octant scores were *z*-transformed because population norms were not available for the here used measure of interpersonal problems.

### Measures

Most of the instruments used in this investigation were constructed and validated for clinical populations (except for the Big-Five-Inventory 2 that assesses normal-range variation in personality; Soto & John, 2017). Apart from the Structured Clinical Interview for *DSM–IV* (*Diagnostic and Statistical Manual for Mental Disorders-Fourth Edition*; First, Spitzer, Gibbon, & Williams, 1997) and the Structured Interview for *DSM–IV* Personality (Pfohl, Blum, & Zimmerman, 1997) all instruments were administered as self-report. Whereas interpersonal problems were measured by all samples, each data set yielded its own distinct pool of external variables.

#### The Inventory of Interpersonal Problems (IIP)

Interpersonal problems were measured with different versions and translations of the IIP (Alden et al., 1990), as indicated in the sample descriptions in Table 1. Measures of interpersonal problems assess problematic interpersonal behaviors that are performed excessively or inhibited strongly. With regard to the IIP, the distress associated with such behaviors is rated, ranging from *not at all* (0) to *extremely* (4) on a 5-point scale. Items are aggregated to obtain octant scores named *Domineering/Controlling* (e.g., “I am too aggressive towards other people”), *Vindictive/Self-Centered* (e.g., “It is hard for me to feel good about another person’s happiness”), *Cold/Distant* (e.g., “It is hard for me to feel close to other people”), *Socially Inhibited* (e.g., “It is hard for me to introduce myself to new people”), *Nonassertive* (e.g., “It is hard for me to confront people with problems that come up”), *Overly Accommodating* (e.g., “I let other people take advantage of me too much”), *Self-Sacrificing* (e.g., “I am overly generous to other people”), and *Intrusive/Needy* (e.g., “I open up to people too much”).

#### The Circumplex Scales of Interpersonal Problems (CSIP)

The CSIP (Boudreaux et al., 2018) consists of 64 items that are rated on a 4-points Likert-type scale. Respondents indicate to what degree the given statements (e.g., “Bossing around other people too much”) are experienced as a problem, ranging from *not a problem* (0) to *serious problem* (3). The CSIP assesses the interpersonal octants, as described earlier. The measure was recently introduced as an alternative instrument to the IIP and demonstrated very good convergent and discriminant validity with its counterpart, although having marginal overlap in wording and content.

#### Symptom-Checklist-90-Revised (SCL-90–R) and Brief Symptom Inventory (BSI)

We used the German translation for the SCL-90–R (Schmitz et al., 2000) and the BSI (i.e., short form of the SCL-90–R; Derogatis, 1993) to assess psychological distress in terms of symptom severity on a 5-point scale ranging from *not at all* (0) to *extremely* (4). The scales include *Somatization* (e.g., “Trouble getting your breath”), *Obsessive-Compulsion* (e.g., “Having to check and double-check what you do”), *Interpersonal Sensitivity* (e.g., “Others are unsympathetic”), *Depression* (e.g., “Feeling Blue”), *Anxiety* (e.g., “Heart pounding/racing”), *Hostility* (e.g., “Urges to harm someone”), *Phobic Anxiety* (e.g., “Afraid on the street”), *Paranoid Ideation* (e.g., “Having beliefs that others do not share”), and *Psychoticism* (e.g., “You should be punished for your sins”). The *General Severity Index* (GSI) is the mean from all subscales and captures global symptom severity (Urbán et al., 2014).

#### Barratt’ Impulsiveness Scale (BIS-11)

The BIS-11 (Patton, Stanford, & Barratt, 1995) measures impulsiveness with 30 items, assessing the frequency of impulsive behavior on a 4-point scale ranging from *rarely/never* (1) to *almost always/always* (4). Our study used only the *Attentional Impulsiveness* scale, which denotes the inability to focus or concentrate (e.g., “I often have extraneous thoughts when thinking”).

#### Empathy Quotient (EQ)

The EQ (Baron-Cohen & Wheelwright, 2004) contains 40-items (and 20 filler items) to measure empathy, as defined by the ability to perceive and understand the intentions of others. The instrument showed a multidimensional factor structure including *Cognitive Empathy* (e.g., “I can tell if someone is masking their true emotions”), *Emotional Reactivity* (e.g., “Seeing people cry doesn’t really upset me”; reversed), and *Social Skills* (e.g., “I find it hard to know what to do in social situations”; reversed). Items are rated on a 4-point scale ranging from *strongly disagree* (0) to *strongly agree* (3).

#### Schizotypal Personality Questionnaire (SPQ)

The SPQ (Raine, 1991) was used to assess Schizotypal Personality by 74 items that relate to the *DSM–III–R* (APA, 1987) diagnostic criteria of Schizotypal Personality Disorder: ideas of reference, excessive social anxiety, odd beliefs or magical thinking, unusual perceptual experiences, odd or eccentric behavior, no close friends, odd speech, constricted affect, and suspiciousness. Schizotypal traits are generally related to discomfort in social interactions and a reduced capacity for interpersonal relations (APA, 2013). Items are rated as present or absent (0 = *No*, 1 = *Yes*). Factor analyses have shown a 3-dimensional structure (Badcock & Dragović, 2006) including *Cognitive-Perceptual Dysfunction* (e.g., “I sense some person or force”), *Interpersonal Deficits* (e.g., “I tend to keep in the background”), and *Disorganization* (e.g., “I am an odd, unusual person”).

#### The “Other as Shamer” Scale (OAS)

The OAS (Goss, Gilbert, & Allan, 1994) includes 18 items to assess shame experiences that occur from perceived negative evaluations by others. Items (e.g., “Others are critical or punishing when I make a mistake”) are rated on a 5-point frequency scale ranging from *never* (0) to *almost always* (4). The construct has shown a three-factor structure (*Being Seen as Inferior*, *Being Seen as Empty or Trivial*, and *Being Observed Doing Mistakes*). Shame is associated with aggression/hostility or withdrawal from social interactions (Smart Richman & Leary, 2009).

#### Difficulties in Emotion Regulation Strategies Scale (DERS)

The DERS (Gratz & Roemer, 2004) assesses dysregulation of mainly negative emotional states among adults by 36 items on a 5-point scale (1 = *almost never*, 5 = *almost always*). Multidimensional facets include *Nonacceptance of Emotional Responses* (e.g., “When I’m upset, I become angry with myself for feeling that way”), *Difficulty Engaging in Goal-Directed Behavior* (e.g., “When I’m upset, I have difficulties getting work done”), *Impulse Control Difficulties* (e.g., “I experience my emotions as overwhelming and out of control”), *Lack of Emotional Awareness* (e.g., “I pay attention to how I feel”; reversed), *Limited Access to Emotion Regulation Strategies* (e.g., “When I’m upset, I believe that I will remain that way for a long time”), and *Lack of Emotional Clarity* (e.g., “I am confused about how I feel”).

#### Posttraumatic Stress Disorder Checklist (PCL)

The PCL (Blanchard, Jones-Alexander, Buckley, & Forneris, 1996) assesses the 17 symptoms of posttraumatic stress disorder postulated in *DSM–IV*. Symptomatic distress is rated for the last month on a 5-point scale ranging from *not at all* (1) to *extremely* (5). The latent structure of posttraumatic stress disorder has been subject to debates (Blevins, Weathers, Davis, Witte, & Domino, 2015; Elhai et al., 2011). The PCL is assumed to be constituted by four highly correlated factors including *Reexperiencing* (e.g., “Flashbacks”), *Avoidance* (e.g., “Avoiding thoughts of trauma”), *Hyperarousal* (e.g., “Hypervigilance”), and *Dysphoria* (or *Emotional Numbing*, e.g., “Restricted Affect”).

#### Structured Clinical Interview for *DSM–IV* Axis-I disorders (SCID-I)

The SCID-I (First et al., 1997) was used to assess Axis-I mental disorders as operationalized by the *DSM–IV*. Moderate to excellent interrater agreement was reported for Axis I disorders (Lobbestael, Leurgans, & Arntz, 2011).

#### Structured Interview for *DSM–IV* Personality (SIDP-IV)

The SIDP-IV (Pfohl et al., 1997) was administered to assess *DSM–IV* personality disorders (i.e., Axis-II). Good interrater reliability (i.e., intraclass correlation) was reported (Jane, Pagan, Turkheimer, Fiedler, & Oltmanns, 2006).

Mental disorder diagnoses assessed with SCID-I and SIDP-IV were aggregated to dimensional diagnosis counts based on syndrome clusters from (a) the *Hierarchical Taxonomy of Psychopathology* (HiTOP; internalizing-fear, internalizing-distress, externalizing-antagonism, externalizing-disinhibition, thought disorder, and detachment; Kotov et al., 2017) and (b) Axis-I + II disorders from *DSM–5* (APA, 2013). HiTOP related syndromal clusters are based on the empirically observed covariation of mental disorders. Yet, the psychometric properties of diagnoses counts are not clear.

#### Big-Five-Inventory - 2 (BFI-2)

Personality domains and facets were assessed by the BFI-2 (Soto & John, 2017), consisting of 60 items (four items per facet). Respondents endorse short statements on a 5-point, Likert-type scale ranging from *disagree strongly* (1) to *agree strongly* (5). For the current sample, Boudreaux et al. (2018) reported strong internal consistencies and test-retest reliability at the domain level (α = .76–.84) and the facet level (average α was .76, average retest reliability was .73).

#### Personality Inventory for *DSM–5* (PID-5–100)

Maladaptive personality domains and facets were measured using a short form of the PID-5 (Maples et al., 2015), that consists of 100 items (four items per facet). Respondents rate on a 4-point scale whether the presented statements apply to themselves (e.g., “I don’t get as much pleasure out of things as others seem to.”) ranging from *very false or often false* (0) to *very true or often true* (3). Maladaptive personality dimensions achieved strong internal consistencies at the domain level (α = .81–.89) and the facet level (*Mdn* = .81).

### Latent Variable Models

Octant scores served as indicators to fit latent dimensional, categorical, and hybrid models to data collected from four large samples. All samples had acceptable levels of skewness and kurtosis for the octant scales (< |1|). Nonetheless, all models were estimated with maximum likelihood and robust standard errors (MLR). Mean structures were included in all estimated models to permit for direct comparisons. We provide an overview on the different models and their parameterization in the online supplemental materials (see Table S5 see Figures S1–S3 for structural notations).

Latent class analysis (LCA; note this is sometimes referred to as latent profile analyses when dimensional indicators are used, as was done here) was used to estimate fully categorical latent structures. In LCA, the pattern of covariation among the observed variables is presumed to arise from latent classes that are characterized by different patterns of means on the observed variables; in this case, different profiles of octants scores. Individual deviations from the expected pattern are modeled as random error, and such error variances are set to equivalence across classes. In the current investigation, the optimal number of classes in LCA was determined by exploratory analyses. We used four decision heuristics, including the Bootstrapped Likelihood-Ratio Test (BLRT), the Vuong-Lo-Mendel-Rubin Test (VLMR), the small-sample corrected Akaike’s information criterion (AIC_C_; Burnham & Anderson, 2004), and the Bayesian Information Criterion (BIC; Schwarz, 1978). Multiple candidate models were selected when the stopping rules supported different solutions. We limited the extraction of classes to a maximum of 15 and only considered solutions viable when the smallest class comprised at least 5% of the total sample. Those criteria were liberal boundaries that would exclude fundamentally impractical solutions and prevent overfitting.

For the different variants in dimensional models (i.e., more restrictive vs. less restrictive, normal vs. nonnormal latent distribution, purely factorial vs. factor mixture) we specified a set of candidates that were collectively based on three-dimensions, as suggested by past research (Acton & Revelle, 2002). The factorial part is based on specifying agency and communion as orthogonal factors that show a circular pattern of factor loadings. A third, general factor loads equally on all octants. In contrast to common bifactor models, we let the general factor correlate freely with group factors. This has conceptual reasons, namely, that the general factor is regarded as assessing the degree of overall distress, and the group factors the tendency to experience some problems more than others (in other words: the most prevalent interpersonal style). Freeing those correlations allows for the possibility that different problem contents may be associated with different levels of distress (e.g., submissive problems might be more disturbing than agentic problems).

In our most restrictive CFA-PC (also known as the “perfect circumplex” solution; Gurtman & Pincus, 2003) the factor loadings on the group factors are specified in a way that induces a fixed correlational pattern upon octants characterized by two conditions: equal spacing and equal communalities. Adjacent octants are restricted to have equal spacing in between, as is reflected in the factor loadings (i.e., “equal spacing” condition). Style dimensions are restricted to be measured with identical reliability (i.e., “equal communalities” condition). Finally, the latent factor distributions are predicated on the Gaussian distribution (as is customary in standard CFA). The CFA-PC was selected as a candidate model, because it directly corresponds to the commonly used simple scoring procedure for the IPC domains (Locke, 2010):

Agency=PA+(NO×.71)+(BC×.71)−(FG×.71)−(JK×.71)−HI

Communion=LM+(NO×.71)+(JK×.71)−(BC×.71)−(FG×.71)−DE

$$General\;Interpersonal\;Distress=\frac{\left(PA+BC+DE+FG+HI+JK+LM+NO\right)}{8}$$

In our less restrictive CFA-QC (i.e., the “quasi-circumplex” solution), both spacing and communalities are free to deviate from equality. CFA-QC was included as a candidate model, because previous research suggested that the fit of IPC-based models can be significantly improved by relaxing those assumptions, without sacrificing validity (Acton & Revelle, 2002; Gurtman & Pincus, 2000).

We included additional dimensional models that allowed for deviations from normality and retained the strict conditions for a perfect circumplex. This approach can be applied by skew-*t*-CFA (Asparouhov & Muthén, 2016), allowing the estimation of flexible nonnormal distributions for the latent continuous factors (i.e., skewed, *t*-shaped or both). For the skew-*t*-CFA approach, additional parameters comprise multivariate degrees of freedom and factor-specific skew. When the model does not converge, a simpler approach can be used that does not include the *t*-distribution (i.e., skew-CFA) or the skew parameters (i.e., *t*-CFA). In CFA and skew-*t*-CFA individuals are still assumed to stem from one single population.

This is different for the hybrid approach, as implemented by factor mixture modeling, namely, Semi-Parametric Factor Analysis (SP-FA; Hallquist & Wright, 2014). Like in fully dimensional approaches, individuals are presumed to vary in three continuous dimensions (following the perfect circumplex). However, a latent *k*-fold categorical variable identifies locations in the three-dimensional space in which individuals concentrate in clusters (i.e., to form latent classes). Those latent classes are characterized by distinct patterns on the factor-specific means. Each class is assumed to be normally distributed and factor variances are fixed to be equal in all classes. The dimensional part of the hybrid model was confirmatory and deciding upon the optimal number of latent classes was data-driven by consulting the AIC_C_ and BIC. The maximum number of extracted classes was limited to nine and solutions that included latent classes comprising less than 5% of the total sample were not considered. Supported solutions were selected as candidate models. We also considered including nonparametric factor analysis (NP-FA; Hallquist & Wright, 2014) as a second hybrid modeling approach, however, it was not identified.

### Model Evaluation

Structural validity of models was assessed by relative fit indices to compare nonnested models, corrected Akaike’s information criterion (AIC_C_) and the Bayesian information criterion (BIC). Tests of model fit quantify the degree to which the assumption of local independence is valid (i.e., a common assumption of latent variable models). Local independence states that the covariance between indicators ought to be fully explained by the latent variables included in the model, in other words, indicators ought to be uncorrelated conditioned on the latent variables. AIC_C_ and BIC evaluate model fit but differ in the degree to which model parsimony (e.g., the number of free parameters) is weighted (Dziak, Coffman, Lanza, & Li, 2012; Vrieze, 2012). The BIC more heavily penalizes additional parameters (i.e., weighs parsimony more) and, therefore, when the AIC_C_ and BIC disagree in practice, the BIC always favors a more parsimonious model relative to the AIC_C_.

Concurrent validity of candidate models was evaluated as follows: we estimated individual factor scores and class memberships to investigate criterion-oriented validity of competing approaches, which was defined as the utility of the models in predicting conceptually relevant external variables. Those variables were chosen to capture a broad range of psychopathology and personality-related variation. For this purpose, multiple linear regressions were estimated to predict continuous outcomes (e.g., measures of symptom load) from multiple latent scores (i.e., factor scores and/or class memberships). The adjusted coefficient of determination (*R*^2^) was used to estimate the amount of variance explained in external variables. The relative importance of predictors is indicated by the unique variance explained in external variables (Δ*R*^2^), corresponding to the squared part correlation between predictor and criterion. We dummy-coded the categorical variable denoting latent class membership.^3^ We explored possible interactions between latent dimensions, although previous research has indicated that interactions among IPC factors are not often significant (Wilson et al., 2013).

Several goodness-of-fit indices were calculated for factor analytic models: The comparative fit index (CFI), the Tucker-Lewis Index (TLI), the root mean square error of approximation (RMSEA), and standardized root mean square residual (SRMR). We further calculated statistical indices that are useful for the psychometric evaluation of factor analytic models, for which multiple common sources of variance are present, for example, one general factor and two group factors (Rodriguez, Reise, & Haviland, 2016).^4^ We calculated *Explained Common Variance* (ECV) to estimate the degree to which the octant scores have one single common source of variance, such that the measure could be considered essentially unidimensional. ECV reflects the percent of common variance that can be attributed to the general factor with values closer to 1 indicating stronger unidimensionality. Coefficient *H* was calculated to estimate the degree to which latent factors would likely replicate across samples. Values of *H* greater than .60 are recommended. Factor determinacy (FD) was calculated to evaluate whether factor score estimates could be considered trustworthy to reflect true individual differences. Values of *FD* greater than .90 indicate trustworthy factor scores. Standardized residuals between the model-implied and the observed covariance matrices were investigated to identify localized areas of strain (i.e., misfit), with values greater than |2.56| indicating local misfit.

### Software Packages

Statistical analyses were executed using R (R Core Team, 2017), the Lavaan Package (Rosseel, 2012), the MplusAutomation Package (Hallquist & Wiley, 2018), and Mplus Version 8 (Muthén & Muthén, 1998–2017).

We estimated LCA and SP-FA models beginning with 500 random starts and doubling the number of random starts when needed to replicate the log-likelihood at least 10 times. Multiple regressions with continuous outcome were estimated by Ordinary Least Squares (OLS). Robust regression analysis was employed by the *Robustbase* Package (Maechler et al., 2016) to screen for discrepancies with OLS estimates.

## Results

### Model Estimation

LCA models for up to 15 latent classes were estimated and mostly converged (nonconvergence occurred in Sample 3 for *k* = 15 and in Sample 4 for *k* ≥ 8). The entropy statistic never went below .811 indicating acceptable class separation. Categorical solutions were not considered for subsequent analytic steps when the smallest latent class comprised less than 5% of the total sample, which was the case in Sample 1 for *k* ≥ 10, Sample 2 *k* ≥ 9, Sample 3 *k* ≥ 7, Sample 4 *k* ≥ 5. VLMR favored less complex class solutions, generally (Sample 1 *k* = 5, Sample 2 and 3 *k* = 3, Sample 4 *k* = 2). BLRT, AIC_C_, and BIC favored the most complex solutions, respectively (Sample 1 *k* = 9, Sample 2 *k* = 8, Sample 3 *k* = 6, Sample 4 *k* = 4). Notably, the optimal number of classes was not robust between decision criteria and not robust between samples. Further details on the estimated LCA models can be found in the online supplemental materials (Tables S6–S9).

The most restrictive CFA-PC model (i.e., equal spacing and communalities, latent normal distributions) yielded acceptable fit to the data, CFI = .938–.957, TLI = .928–.950, SRMR = .059–.078 (Samples 1–4). The RMSEA suggested shortcomings in fit, RMSEA = .075–.111, *p* < .001. Across samples, standardized factor loadings were statistically significant, λ_GENERAL_ = .57–.82, λ_AGENCY_ = |.56|–|.64|, λ_COMMUNION_ = |.49|–|.54| (highest loading for the marker octants). *ECV* indicated that the general factor accounted for roughly 75% (and the group factors for 25%) of the common variance, *ECV* = .69–.76 (Samples 1–4). This shows that, although the general factor was the dominant source of variance, the construct should not be considered essentially unidimensional. Coefficient *H* indicated that the general factor was defined well and the style dimensions were defined moderately well by the indicators used, *H*_GENERAL_ = .88–.89, *H*_AGENCY_ = .50–.63, *H*_COMMUNION_ = .49–.58 (Samples 1–4), such that those latent variables would likely replicate across samples. Factor determinacy indicated that factor scores could be trusted to reflect true individual variation, *FD*_GENERAL_ = .96–.97, *FD*_AGENCY_ = .87–.92, *FD*_COMMUNION_ = .85–.90 (Samples 1–4). The latent correlation between agency and the general factor was estimated to be negative (Sample 1 *r* = −.283, Sample 2 *r* = −.292, Sample 3 *r* = −.267, Sample 4 *r* = −.324). The statistical association between communion and the general factor was dependent from the sample investigated (Sample 1 *r* = −.034, Sample 2 *r* = .142, Sample 3 *r* = .083, Sample 4 *r* = −.115). The less restrictive CFA-QC (i.e., allowing for unequal spacing and communalities) produced virtually identical parameter and fit estimates. Therefore, it is not described any further.

Skew-*t*-CFA converged exclusively in Sample 1. Nonnormal skew-*t*-distributions were estimated to have 20.85 *df* and substantial skew for the latent factors, skew_GENERAL_ = 1.39, skew_AGENCY_ = 0.85, skew_COMMUNION_ = −0.89. Skew-normal-CFA did not converge in Samples 2–4. We estimated *t*-distributed CFA models without skew parameter in the samples remaining, *df* = 8.43 (Sample 2), *df* = 13.78 (Sample 3), *df* = 6.35 (Sample 4). Standardized residuals between the model-implied and the observed covariance matrices showed no significant localized areas of misfit for Samples 1–3 (i.e., IIP), ranging from −0.27 to 0.22. In Sample 4, standardized residuals ranged from −3.10 to 1.76 indicating local misfit.

Nonconvergence of SP-FA solutions occurred in Sample 2 and Sample 4 (for *k* ≥ 5). Hybrid solutions were not considered when the smallest latent class comprised less than 5% of the total sample, which was the case in Sample 1 for *k* ≥ 6, Sample 3 *k* ≥ 3, Sample 4 *k* = 4. AIC_C_ and BIC univocally selected the most complex hybrid solutions available (Sample 1 *k* = 5, Sample 2 *k* = 4, Sample 3 *k* = 2, Sample 4 *k* = 3). Further details, including SP-FA model’s entropy statistics and smallest class proportions, are described in the online supplemental materials (Tables S10–S13).

### Model Comparison

Direct comparisons between dimensional, hybrid, and categorical candidate models are summarized in Table 2. Smaller values of AIC_C_ and BIC indicate favorable fit. Higher values in *R*^2^ are preferable, as more variance is explained by model-based factor scores and/or predicted class memberships. Further details into model performance by sample are available in the online supplemental materials (Tables S14–S17).[Table-anchor tbl2]

With regard to dimensional models in this study, relaxing the “equal spacing and equal communalities” restriction did not result in consistent improvements in terms of model fit. In contrast, relaxing the restriction of latent normality by means of skew-*t*-CFA and *t*-CFA resulted in greatly improved model fit. However, it did not increment the prediction of external variables when compared against the more simplistic CFA model. Visual inspection of the density plots revealed one explanation for this finding: The distribution of estimated factor scores had almost identical shape for all models that included the three dimensions (i.e., CFA-PC, nonnormal CFA, SP-FA; see online supplemental materials, Figures S4–S7). Correlations between factor scores were consistently greater than .95, demonstrating that those scores carried the same information independently of specification.

Model fit favored the dimensional models. The BIC and AIC_C_ univocally selected *t*-distributed CFA models in Samples 1, 3, and 4 (the number of free parameters for those CFA models were κ = 21 and κ = 24). In Sample 2, BIC and AIC_C_ selected the hybrid SP-FA model, κ = 32. However, this might have to do with the bimodal distribution observed in Sample 2, because one subset (i.e., outpatients) was selected for extreme values and the other subset was not (i.e., control group).^5^ Variance explained in external variables favored dimensions, while fully categorical models showed poor performance in predicting external variables. Within fully categorical models, variance explained was greater for more complex solutions. Notably, hybrid models and fully dimensional models performed equally well in predicting external variables. However, critically, estimated class memberships in SP-FA models did not increment the prediction after accounting for the variance explained by factor scores. All things considered (i.e., model fit, variance explained in external variables, consistency across samples), the best performance was achieved by fully dimensional models that permit for nonnormal factor distributions (i.e., skew-*t*-CFA, *t*-CFA model).

We report on the associations observed between factor scores from CFA-PC and external variables in Table 3, to evaluate the relevance of interpersonal problems in relation to diverse pathological and personality-related outcomes. Predictive utility of interpersonal dimensions was considered substantial for values of Δ*R*^2^ greater than .05. The general factor accounted for the largest share of variance explained in external variables (however, those were mostly markers of symptomatic distress). Agency and communion showed substantial and consistent associations. An agentic style was associated with extraversion, hostility, antagonism, paranoid ideation, disinhibition, low empathy, and low agreeableness. An affiliative style was associated with extraversion, low detachment, empathy, and agreeableness. The predictive utility of interactions was trivial, except for predicting the agreeableness domain. Associations between interpersonal dimensions and HiTOP-related diagnosis counts were less pronounced in the current study (yet, the psychometric properties of diagnosis counts are not clear). Variance explained was largest for the bimodal sample, indicating that the dimensional factor scores performed well in differentiating between outpatients and the healthy control group.[Table-anchor tbl3]

## Discussion

In the current study, we compared dimensional, categorical, and hybrid models of interpersonal problems considering structural and concurrent validity. Across four samples we found consistent support for the superior validity of a purely dimensional representation (i.e., confirmatory factor analytic models based on the IPC), especially when allowing for nonnormal latent distributions. No evidence was found for the incremental validity of categorical or hybrid approaches.

### Dimensions Versus Types

Dimensional models outperformed fully categorical models with regard to fit indices, showing that the covariance between octant scores was more accurately reproduced by three dimensions than by any number of latent classes or hybrid models. Also, the criteria used to explore the optimal number of classes for LCA models did not generate consistent and replicable results. The VLMR selected sparse LCA solutions, whereas the other stopping criteria selected the most complex class solutions available. In most cases the number of suggested classes is difficult to conceptualize or apply in practical work. Furthermore, the dimensional models outperformed purely categorical models in terms of prediction. Most likely, the latent classes are artificial, because fitting categorical models to a truly dimensional latent structure can result in extracting spurious classes (Lubke & Neale, 2006). Such spurious classes would partially cover the dimensional variance, in that the variance explained increases as a function of classes extracted. Our results indicate that this was the case in the present study. With regard to the hybrid approach, the extracted latent classes did not increment the prediction (i.e., after controlling for factor scores). Also, the shapes of the density distributions did not convey the impression that individuals concentrated in distinct clusters. Considering our results, the concordance in shape between the hybrid and fully dimensional approach appears to be more consistent with having nonnormal population distributions. This inherent nonnormality could fully account for the formation of classes in hybrid models. Thus, the additional classes may simply compensate for deviations from the normal assumption (Bauer & Curran, 2003) without providing any substantial information. In light of the evidence presented here, it seems questionable to assume that actual discrete interpersonal subpopulations might exist. Although more evidence will be needed to substantiate this claim, our results seem to be robust with respect to reproducibility and generalizability: They are based on four larger samples of individuals from different countries and cultural backgrounds spanning community recruited participants to patients with severe personality problems using two measures of interpersonal problems.

If the latent structure of interpersonal problems was truly dimensional, categorical scoring of interpersonal problems might result in loss of statistical power (Markon et al., 2011; Morey et al., 2012; Preacher et al., 2005) and impede statistical inferences (Kane, 2013). For illustration, imagine a latent class that was characterized by an octant score profile pattern aligning with high distress and a dominance-related style. It would be indistinguishable whether associations of class membership to other constructs would have resulted from severity or style. For example, Cain et al. (2012) reported six clusters from which the submissive type predicted chronicity of major depression. Closer inspection reveals that chronicity was greatest for the submissive class, but also high for other low agency classes. Based on our findings, we could reframe the authors’ conclusion to say that low agency might have predicted chronicity (instead of class membership). Yet, based on the report we cannot retain a precise point estimate and confidence intervals for the effect, because the categorical approach neglects within-class variance and treats it as random error, artificially shrinking the effect. Another concern is that general distress might have driven the effect, because multidimensional variation was confounded within the classes reported (and because severity appears to be correlated with style). This example shows that inferences based on categorical scoring (i.e., cluster analysis, latent class/profile analysis) might be significantly curtailed, given that the latent structure of interpersonal problems was multidimensional. Still, empirical results from those approaches can be interpreted with reference to IPC-based dimensions, as was demonstrated here.

### Relevance of Interpersonal Dimensions

The common variance among general interpersonal distress and clinical measures relates to a general factor of psychopathology (Caspi & Moffitt, 2018), indicating that self-reported interpersonal problems include a generic form of symptomatic distress (Tracey et al., 1996). According to one hypothesis, general interpersonal distress reflects diffuse interpersonal impairments that lead to real-world consequences for the individual. Such impairments have recently been conceptualized as underpinned and maintained by a particular lack of resilience, namely a complex interplay between biological factors, maltreatment, impaired mentalizing, and epistemic mistrust that compromises social learning (Fonagy & Allison, 2014; Nolte, Campbell, & Fonagy, 2019). A second hypothesis is that general interpersonal distress mainly reflects distorted cognition (i.e., dissatisfaction with interpersonal relations). Multimethod approaches could provide further insights into the nature of this factor.

Concerning the IPC-related style dimensions, our results substantiate the notion that high agency aligns robustly with antagonistic personality (Williams & Simms, 2016) and the externalizing symptom spectrum in HiTOP (Kotov et al., 2017). Our study indicates that agentic problems might be less associated with overall distress, mirroring earlier findings (Wright et al., 2012). Unsurprisingly, low communion seems to align with the detachment trait and symptom spectrum (Kotov et al., 2017). Besides, lacking associations between interpersonal styles and many psychopathologies deserve further attention, in that, most prominently, depression and anxiety (i.e., two main areas of pathoplasticity research) could not be related to specific interpersonal styles. Yet, interpersonal styles were indeed strongly associated with normative personality traits at the domain level (i.e., extraversion, agreeableness) and the facet level (e.g., assertiveness, sociability, and compassion), suggesting that interpersonal styles might largely reflect nonpathological dispositions of interacting with others. Prior research has indicated that “cold” problems were better covered by maladaptive traits and “warm” problems were better covered by normative traits (Williams & Simms, 2016; Wright et al., 2012). As a general conclusion, we suggest that variation in interpersonal styles is likely to result from both pathological processes and temperamental differences. Longitudinal investigations could procure further insights into the causal pathways.

### Limitations

The current investigation was based on self-report measures of interpersonal functioning. However, correlations between self-reports of IIP and informant-reports of impact messages (Altenstein-Yamanaka et al., 2017; Quilty et al., 2013) or social competences (Leising, Krause, Köhler, Hinsen, & Clifton, 2011) were rather small. This raises the question whether the structure is dependent on the assessment method (self-report, assessment by mental health professionals, or significant others) and also, which method would deliver the most valuable information. Another limitation of our study is that the most adequate model might also be dependent on the populations investigated (e.g., Eaton, Krueger, South, Simms, & Clark, 2011). Lastly, our study did not allow for evaluation of predicting future outcomes (e.g., therapy outcome measures).

### Conclusions and Practical Recommendations

A longstanding tradition has modeled interpersonal dispositions in a circumplex (e.g., Alden et al., 1990; Horowitz et al., 2006; Kiesler, 1983; Leary, 1958; Wiggins, 1979). However, the coexistence of dimensional and categorical approaches to score interpersonal problems impeded the conceptual and statistical integration of empirical results. Although the categorical interpretation (i.e., prototype model) might be pragmatic for practitioners and patients, we found little evidence that latent classes can enhance the conception of interpersonal problems. The latent structure of interpersonal problems was best described by IPC-based continuous dimensions, especially when allowing for nonnormal latent distributions. To date, most other constructs in psychopathology research have also shown a dimensional structure (Aslinger, Manuck, Pilkonis, Simms, & Wright, 2018; Carragher et al., 2014; Haslam, Holland, & Kuppens, 2012; Wright et al., 2013b).

For future investigations and meta-analyses, we recommend to use a unitary modeling approach for interpersonal research to advance cumulative science. We believe that our results support the construct validity of dimensional IPC-based scores (see Method section for scoring formulas, see online supplemental material R Codes S1–S3 for latent variable applications to use in statistical software). The IPC-based approach offers a parsimonious model that is easy to interpret and to implement (i.e., standard scoring), useful for prediction purposes, and it does not require model estimation (i.e., no sample size requirements, no risk of overfitting). Another benefit is that the IPC is embedded in a metaframework (Dawood et al., 2018; Pincus, Lukowitsky, & Wright, 2010) that provides a link to motivational (being in control of and being close to others) and behavioral aspects of personality (dominance and nurturance). We highlight the importance of including all three interpersonal dimensions in regression analyses to account for correlations between them and to establish the incremental information of severity (i.e., general distress) and style (i.e., agency and communion). We advise against the use of categorical scoring procedures for heuristic purposes for the stated reasons. One area of work that has most relied on those techniques is pathoplasticity research (e.g., Cain et al., 2010, 2012; Przeworski et al., 2011; Wright et al., 2013), with a consistent finding that certain disorders encompass many diverse interpersonal “types” that provide incremental clinical information above diagnosis. How should this research proceed without enlisting categorical interpersonal models? We argue that intradiagnosis interpersonal heterogeneity will be reflected in high variability (e.g., *SD*s) of interpersonal style dimensions within diagnostic groups, as well as in low associations between interpersonal style dimensions and the pathology in question. When such high variability and low correlations occur, they can be further investigated with visual plots (as is common in pathoplasticity research) and the incremental validity of the style dimensions can be tested using standard procedures. Notably, depression and anxiety pathologies form main topics in pathoplasticity research, and for those, no substantial associations with interpersonal styles were found in the current study.

We further advise against the use of octant scores, because the IPC-based model yields a parsimonious summary with sufficient approximation to diverse clinical populations. Future structural analyses of psychological constructs should consider nonnormal approaches (when practically feasible for a given sample size) to avoid being misled into retaining hybrid solutions. Furthermore, our results underline that hybrid solutions should be evaluated on the grounds of both structural and criterion-oriented validity.

The interpersonal sphere denotes an important domain of personality functioning. We examined the latent structure of interpersonal problems and illustrated the relevance of interpersonal dimensions for psychopathology research by enlisting associations to clinical symptom markers and personality-related variables. Our study provides guidance and practical recommendations for future investigations to study interpersonal problems and their correlates, including a call for a unitary use of the IPC-based dimensional model. Topics for future research may include the moderating effects of interpersonal style on treatment outcome (i.e., personalized psychotherapy research) or investigating the changes in interpersonal style in the course of mental illness (i.e., pathoplasticity research).

## Supplementary Material

10.1037/abn0000460.supp

## Figures and Tables

**Table 1 tbl1:** Samples and Measures

No.	Sample description	*N*	Age *M* (*SD*)	Interpersonal measure	External criterion variables	
1	Treatment-seeking individuals, Germany (Benecke et al., 2011; Henkel et al., 2017)	5,400	37.4 (11.8)	IIP-64, German translation (Horowitz, Strauss, & Kordy, 2000), octant scales internal consistencies ω =.71–.87, standardized by population norms	Symptom Checklist 90 Revised (SCL-90-R; Schmitz et al., 2000)	General Severity Index
						Somatization
						Obsessive-compulsiveness
						Interpersonal sensitivity
						Depression
						Anxiety
						Hostility
						Paranoid ideation
						Phobic anxiety
						Psychoticism
2	Outpatients and healthy controls, United Kingdom (Euler et al., 2019)	491	31.5 (10.7)	IIP-32 (Horowitz, Alden, Wiggins, & Pincus, 2000), octant scales’ internal consistencies ω = .66–.85, standardized by population norms	Brief Symptom Inventory (BSI; Derogatis, 1993)	General Severity Index
					Baratt Impulsiveness Scale (BIS-11; Patton, Stanford, & Barratt, 1995)	Somatization
					Empathy Quotient (EQ; Baron-Cohen & Wheelwright, 2004)	Obsessive-compulsiveness
					The Other as Shamer Scale (OAS; Goss, Gilbert, & Allan, 1994)	Interpersonal sensitivity
					Difficulties in Emotion Regulation Scale (DERS; Gratz & Roemer, 2004)	Depression
					Schizotypal Personality Questionnaire (SPQ; Raine, 1991)	Anxiety
					Posttraumatic Stress Checklist Scale; (Blanchard, Jones-Alexander, Buckley, & Forneris, 1996)	Hostility
						Paranoid ideation
						Phobic anxiety
						Psychoticism
						Cognitive impulsiveness
						Empathy
						External shame
						Emotional dysregulation
						Schizotypal personality
						Posttraumatic stress
3	Clinical and community samples subsetted for individuals that meet one *DSM-5* mental disorder diagnosis, United States; Girard et al. (2017)	656	36.0 (10.5)	IIP-64 (Alden, Wiggins, & Pincus, 1990), octant scales’ internal consistencies α = .76–.90, standardized by population norms	The Structured Clinical Interview for *DSM-IV* Axis I Disorders (SCID-I; First, Spitzer, Gibbon, & Williams, 1997) and The Structured Interview for *DSM-IV* Personality (SIDP-IV; Pfohl, Blum, & Zimmerman, 1997)	Total diagnoses count
						Axis-I diagnoses
						Axis-II diagnoses
						Internalizing-fear
						Internalizing-distress
						Externalizing-antagonism
						Externalizing-disinhibition
						Detachment
						Thought disorder
4	Undergraduate students at the University of Pittsburgh, United States; Boudreaux, Ozer, Oltmanns, and Wright (2018)	712	18.7 (1.7)	CSIP (Boudreaux et al., 2018), octant scales’ internal consistencies α = .78–.89 *z*-transformed	Big-Five-Inventory−2 (BFI-2; Soto & John, 2017)	Negative emotionality
					Personality Inventory for *DSM-5* (PID-5–100; Maples et al., 2015)	Agreeableness
						Conscientiousness
						Extraversion
						Open-mindedness
						Negative affect
						Antagonism
						Disinhibition
						Detachment
						Psychoticism
*Note*. *DSM-5* = *Diagnostic and Statistical Manual for Mental Disorders-Fifth Edition*; IIP = Inventory of Interpersonal Problems; CSIP = Circumplex Scales of Interpersonal Problems; α = Cronbach’s α; ω = McDonald’s omega.						

**Table 2 tbl2:** Summary of Model Fit and Variance Explained in External Variables for Dimensional, Categorical, and Hybrid Candidate Models of Interpersonal Problems Across Samples

Sample	κ	Factors	Classes	Statistical model	AIC_C_	BIC	Median *R*^2^
								Range of Δ*R*^2^			
								General distress	Agency	Communion	Class membership
Sample 1 (*N* = 5,400)	20	3		CFA-PC	111,545	111,413	**.32**	.10–.40	.00–.08	.00–.02	
	23	3		CFA-QC	111,496	111,344	**.32**	.10–.40	.00–.08	.00–.02	
	24	3		Skew-*t*-CFA	**110,337**	**110,495**	**.32**	.10–.40	.00–.08	.00–.02	
	36	3	5	SP-FA^a,b^	110,438	110,674	**.32**	.04–.14	.00–.04	.00–.01	.00–.00
	52		5	LCA^c^	118,573	118,915	.27				.09–.40
	88		9	LCA^a,b,d^	114,771	115,348	.28				.09–.42
Sample 2 (*N* = 491)	20	3		CFA-PC	12,545	12,463	**.46**	.12–.49	.00–.10	.00–.11	
	23	3		CFA-QC	12,547	12,453	**.46**	.12–.49	.00–.10	.00–.11	
	21	3		*t*-CFA	12,418	12,505	**.46**	.12–.50	.00–.10	.00–.11	
	32	3	4	SP-FA^a,b^	**12,305**	**12,435**	.45	.02–.12	.00–.02	.00–.07	.00–.01
	34		3	LCA^c^	12,910	13,047	.33				.03–.45
	79		8	LCA^a,b,d^	12,361	12,662	.39				.12–.51
Sample 3 (*N* = 656)	20	3		CFA-PC	13,622	13,710	**.13**	.01–.18	.00–.09	.00–.06	
	23	3		CFA-QC	13,719	**13,617**	**.13**	.01–.18	.00–.09	.00–.06	
	21	3		*t*-CFA	**13,525**	**13,617**	**.13**	.01–.18	.00–.09	.00–.06	
	24	3	2	SP-FA^a,b^	13,575	13,681	**.13**	.01–.13	.00–.06	.00–.06	.00–.00
	34		3	LCA^c^	15,024	15,173	.07				.00–.12
	61		6	LCA^a,b,d^	14,446	14,707	.07				.00–.15
Sample 4 (*N* = 712)	20	3		CFA-PC	30,271	30,181	**.25**	.01–.23	.00–.29	.00–.15	
	23	3		CFA-QC	30,262	30,159	**.25**	.01–.23	.01–.29	.00–.15	
	21	3		*t*-CFA	**29,831**	**29,926**	**.25**	.01–.22	.00–.29	.00–.15	
	28	3	3	SP-FA^a,b^	29,908	30,034	**.25**	.00–.12	.00–.17	.00–.12	.00–.02
	25		2	LCA^c^	31,968	32,080	.08				.00–.15
	43		4	LCA^a,b,d^	31,089	31,279	.15				.00–.23
*Note*. Most favorable values are highlighted in bold print. κ = number of free parameters; AIC_C_ = corrected Akaike’s information criterion; BIC = Bayesian information criterion; Median *R*^2^ = average explained variance in external variables by sample; Range of Δ*R*^2^ = range of unique variance explained in external variables by sample; CFA-PC = confirmatory factor analysis (perfect circumplex); CFA-QC = confirmatory factor analysis (quasi circumplex); Skew-*t*-CFA = confirmatory factor analysis with non-normal latent skewed *t*-distribution; SP-FA = semiparametric factor analysis. LCA = Latent class analysis; VLMR = Vuong-Lo-Mendel-Rubin Test; BLRT = Bootstrapped Likelihood-Ratio Test.											
^a^ Optimal number of classes by AIC_C_. ^b^ Optimal number of classes by BIC. ^c^ Optimal number of classes by VLMR. ^d^ Optimal number of classes by BLRT.											

**Table 3 tbl3:** Unique Contributions of Factor Scores (3-Factor CFA-PC) in Predicting External Variables

				Δ*R*^2^						
Sample	Measure		Predicted external variable	General distress		Agency		Communion		Interactions
Sample 1 (*N* = 5,400)	SCL-90-R		General Severity Index	**.39**	**(+)**	.00		.00		.01
			Somatization	**.16**	**(+)**	.00		.00		.00
			Obsessive-compulsiveness	**.28**	**(+)**	.00		.00		.00
			Interpersonal sensitivity	**.40**	**(+)**	.00		.00		.01
			Depression	**.29**	**(+)**	.00		.00		.00
			Anxiety	**.20**	**(+)**	.00		.00		.00
			Hostility	**.27**	**(+)**	**.08**	**(+)**	.00		.00
			Paranoid ideation	**.37**	**(+)**	.00		.00		.01
			Phobic anxiety	**.12**	**(+)**	.00		.01		.01
			Psychoticism	**.33**	**(+)**	.01		.01		.00
Sample 2 (*N* = 491)	BSI		General Severity Index	**.48**	**(+)**	.01		.00		.01
			Somatization	**.27**	**(+)**	.01		.00		.02
			Obsessive-compulsiveness	**.34**	**(+)**	.00		.00		.01
			Interpersonal sensitivity	**.42**	**(+)**	.00		.00		.01
			Depression	**.38**	**(+)**	.00		.00		.01
			Anxiety	**.36**	**(+)**	.01		.00		.01
			Hostility	**.36**	**(+)**	**.10**	**(+)**	.00		.00
			Paranoid ideation	**.34**	**(+)**	**.05**	**(+)**	.00		.01
			Phobic anxiety	**.37**	**(+)**	.00		.00		.02
			Psychoticism	**.46**	**(+)**	.01		.00		.01
	BIS		Cognitive impulsiveness	**.33**	**(+)**	.00		.00		.01
	EQ		Empathy	**.12**	**(−)**	**.05**	**(−)**	**.11**	**(+)**	.02
	OAS		External shame	**.46**	**(+)**	.00		.00		.01
	DERS		Emotional dysregulation	**.45**	**(+)**	.00		.00		.01
	SPQ		Schizotypal personality	**.49**	**(+)**	.01		.01		.01
	PCL		Posttraumatic stress	**.44**	**(+)**	.04		.00		.02
Sample 3 (*N* = 656)	SCID-II/SIDP-IV diagnosis counts		Total diagnoses	**.18**	**(+)**	.00		.00		.01
			Axis-I	**.07**	**(+)**	.00		.00		.00
			Axis-II	**.14**	**(+)**	.02		.00		.01
			Internalizing-fear	.02		.03		.01		.01
			Internalizing-distress	**.12**	**(+)**	.00		.00		.01
			Externalizing-antagonism	**.11**	**(+)**	**.07**	**(+)**	.00		.01
			Externalizing-disinhibition	.01		**.07**	**(+)**	.00		.00
			Detachment	.04		**.09**	**(−)**	**.06**	**(−)**	.01
			Thought disorder	.01		.01		.02		.01
Sample 4 (*N* = 712)	PID-5-100 Domains		Negative affect	**.18**	**(+)**	.01		.01		.01
			Antagonism	**.20**	**(+)**	**.18**	**(+)**	.00		.03
			Disinhibition	**.16**	**(+)**	.01		.00		.02
			Detachment	**.12**	**(+)**	.00		**.14**	**(−)**	.01
			Psychoticism	**.12**	**(+)**	.03		.01		.01
	BFI-2 Domains		Negative emotionality	**.13**	**(+)**	.02		.00		.01
			Agreeableness	**.17**	**(−)**	**.17**	**(−)**	**.09**	**(+)**	**.06**
			Conscientiousness	**.06**	**(−)**	.00		.00		.02
			Extraversion	.02		**.27**	**(+)**	**.15**	**(+)**	.02
			Open-mindedness	.01		.00		.00		.00
	BFI-2 Facets	N	Anxiety	**.06**	**(+)**	.03		.00		.01
			Depression	**.12**	**(+)**	.03		.02		.01
			Emotional volatility	**.11**	**(+)**	.00		.01		.01
		A	Compassion	**.07**	**(−)**	**.13**	**(−)**	**.13**	**(+)**	**.05**
			Respectfulness	**.16**	**(−)**	**.21**	**(−)**	.02		.04
			Trust	**.12**	**(−)**	.04		**.05**	**(+)**	.03
		C	Organization	.01		.00		.00		.02
			Productiveness	**.05**	**(−)**	.01		.00		.02
			Responsibility	**.08**	**(−)**	.01		.00		.01
		E	Sociability	.01		**.20**	**(+)**	**.21**	**(+)**	.03
			Assertiveness	.00		**.32**	**(+)**	.01		.03
			Energy level	.04		.04		**.13**	**(+)**	.02
		O	Intellectual curiosity	.00		.01		.00		.00
			Aesthetic sensitivity	.00		.01		.00		.00
			Creative imagination	.01		.02		.00		.02
	PID-5–100 Facets	NEG	Emotional lability	**.14**	**(+)**	.00		.01		.01
			Anxiousness	**.12**	**(+)**	.02		.00		.00
			Separation insecurity	**.10**	**(+)**	.00		.03		.00
		ANT	Manipulativeness	**.13**	**(+)**	**.19**	**(+)**	.00		.02
			Deceitfulness	**.22**	**(+)**	**.08**	**(+)**	.00		.02
			Grandiosity	**.10**	**(+)**	**.13**	**(+)**	.01		.02
		DIS	Irresponsibility	**.15**	**(+)**	.02		.00		.01
			Impulsivity	**.08**	**(+)**	**.05**		.01		.01
			Distractibility	**.05**	**(+)**	.01		.01		.02
		DET	Withdrawal	**.13**	**(+)**	.00		**.14**	(**−**)	.02
			Anhedonia	**.13**	**(+)**	.00		**.07**	(**−**)	.02
			Intimacy avoidance	.01		.01		**.05**	(**−**)	.01
		PSY	Unusual beliefs and experiences	**.05**	**(+)**	.04		.00		.01
			Eccentricity	**.11**	**(+)**	.03		.02		.01
			Perceptual dysregulation	**.08**	**(+)**	.01		.01		.01
*Note*. Values of Δ*R*^2^ > .05 were considered substantial and are highlighted in bold print (the key of the association is noted in parentheses). Δ*R*² = unique variance explained in external variables; SCL-90-R = Symptom-Checklist-90-Revised; BSI = Brief Symptom Inventory; BIS = Barratt Impulsiveness Scale; EQ = Empathy Quotient; OAS = The Other as Shamer Scale; DERS = Difficulties in Emotion Regulation Scale; SPQ = Schizotypal Personality Questionnaire; PCL = Posttraumatic Stress Disorder Checklist Scale; SCID-I = Structured Clinical Interview for *DSM–IV* Axis-I disorders; SIDP-IV = Structured Interview for *DSM–IV* Personality; BFI-2 = Big-Five-Inventory 2; PID-5-100 = Personality Inventory for *DSM–5*; N = neuroticism; A = agreeableness; C = conscientiousness; E = extraversion; O = open-mindedness; NEG = negative affectivity; ANT = antagonism; DIS = disinhibition; DET = detachment; PSY = psychoticism.										

## Footnotes

1 Some authors simply used the eight raw octant scores of interpersonal problems (Dammann et al., 2016; Gerhart, Baker, Hoerger, & Ronan, 2014; Quinlan, Deane, Crowe, & Caputi, 2018; Wilson, Stroud, & Durbin, 2017). Others scored the quadrants (König, Onnen, Karl, Rosner, & Butollo, 2016) or cluster analyzed IPC-based scores (Cain et al., 2010).

2 In contrast, regular CFA assumes one single population in which individuals are concentrated in the center of the multivariate normal distribution.

3 However, the coding does not influence how much variance is explained by the polytomous predictor variable.

4 Note that in the current CFA model the general factor is free to correlate with the group factors (unlike in a true bifactor models). Therefore, statistics that are specific to classic bifactor models should not be calculated, because they require unique partitioning of indicator variance.

5 Visual inspection of factor scores revealed that the SP-FA approach was superior in accounting for this inherent bimodality. For reasons of simplicity, we did not account for this bimodality in our candidate models. Yet ideally, one could specify a semiparametric *t*-distributed confirmatory factor model including three *t*-distributed factors and two classes.
