# Supplementary material for: The Latent Structure of Interpersonal Problems: Validity of Dimensional, Categorical, and Hybrid Models
Source: J Abnorm Psychol. 2019 Sep 26;128(8):823–39. doi: 10.1037/abn0000460 (PMC6816327; doi:10.1037/abn0000460)
Supplement: Supplementary file 1 [file ABN-2018-0746_Suppl.zip › latent structure of IP _min_rev_ online supplement a.pdf]

Online Supplementary Material

Table S1

Descriptive Statistics and Bivariate Correlations of Scale Scores in Sample 1 (N = 5400)

							Bivariate Correlations									
		M	*SD*	Skew	Kurtosis	Ω	**1**	**2**	**3**	**4**	**5**	**6**	**7**	**8**	**9**	**10**
(1)	General Interpersonal Distress (IIP-32)[1]	0.20	0.87	–0.07	–0.36	.92	1	.34	.56	.68	.58	.46	.46	.37	.60	.58
(2)	Somatization (SCL-90-R)	0.86	0.69	1.09	0.96	.84		1	.53	.41	.51	.66	.42	.49	.42	.47
(3)	Obsessive-Compulsiveness (SCL-90-R)	1.34	0.76	0.54	–0.18	.84			1	.63	.76	.64	.54	.47	.58	.63
(4)	Interpersonal Sensitivity (SCL-90-R)	1.23	0.79	0.70	0.70	.85				1	.72	.55	.59	.49	.76	.68
(5)	Depression (SCL-90-R)	1.64	0.84	0.20	0.20	.89					1	.63	.58	.45	.60	.67
(6)	Anxiety (SCL-90-R)	1.01	0.74	0.89	0.89	.85						1	.54	.66	.53	.63
(7)	Hostility (SCL-90-R)	0.96	0.77	1.08	1.08	.73							1	.37	.61	.55
(8)	Phobic Anxiety (SCL-90-R)	0.50	0.67	1.96	1.96	.78								1	.42	.53
(9)	Paranoid Ideation (SCL-90-R)	1.02	0.82	0.88	0.88	.78									1	.65
(10)	Psychoticism (SCL-90-R)	0.66	0.57	1.20	1.80	.77										1

Note. Correlations were calculated by Pearson's formula, Ω = Internal consistency, as computed by McDonald's formula. [1] = Standardized by population norms.

Table S2

Descriptive Statistics and Bivariate Correlations of Scale Scores in Sample 2 (N = 491)

						Bivariate Correlations							
	M	*SD*	Skew	Kurtosis	Ω	**1**	**2**	**3**	**4**	**5**	**6**	**7**	**8**
(1) General Interpersonal Distress[1] (IIP-32)	1.02	1.1	–0.13	–0.97	.86	1	.75	.64	–.24	.76	.74	.72	.68
(2) General Severity Index[2] (BSI)	1.4	1.0	0.32	–1.07	.97		1	.68	–.25	.84	.79	.79	.83
(3) Attentional Impulsiveness[2] (BIS)	19.4	5.3	0.05	–0.77	.54			1	–.24	.65	.72	.71	.67
(4) Empathy[2] (EQ)	40.0	13.1	0.19	0.13	.86				1	–.25	–.28	–.34	–.20
(5) External Shame[2] (OAS)	31.6	21.5	0.17	–1.13	.96					1	.79	.78	.74
(6) Difficulties in Emotion Regulation[2] (DERS)	111.5	36.6	–0.15	–1.25	.93						1	.76	.79
(7) Schizotypical Personality[2] (SPQ)	34.1	18.3	–0.10	–1.04	.95							1	.76
(8) Posttraumatic Stress Disorder Symptom Checklist[2] (PCL)	49.7	20.1	–0.12	–1.26	.96								1

Note. Correlations were calculated by Pearson's formula, Ω = Internal consistency, as computed by McDonald's formula. [1] = Standardized by population norms. [2] = Unstandardized.

Table S3

Descriptive Statistics and Bivariate Correlations of Scale Scores in Sample 3 (N = 656)

	M	*SD*	Range	Skew	Kurtosis	Bivariate Correlations									
						**1**	**2**	**3**	**4**	**5**	**6**	**7**	**8**	**9**	**10**
(1) General Interpersonal Distress[1] (IIP-36)	0.77	0.87	−1 – 3.5	0.13	−0.56	1	.43	.31	.36	.23	.37	.27	.04	.29	.06
(2) Total[2] (SCID-II)	2.35	1.67	0 – 10	0.96	1.22		1	.67	.85	.40	.51	.64	.40	.48	.35
(3) Axis-I[2] (SCID-II)	1.04	0.89	0 – 4	0.67	0.12			1	.19	.53	.44	.11	.46	.32	.02
(4) Axis-II[2] (SCID-II)	1.31	1.26	0 – 8	1.08	1.35				1	.16	.36	.77	.20	.41	.45
(5) Internalizing-Fear[2] (SCID-II)	0.31	0.56	0 – 3	1.73	2.50					1	.15	.04	−.06	.47	.01
(6) Internalizing-Distress[2] (SCID-II)	1.14	0.94	0 – 4	0.45	-0.45						1	.39	.00	.15	.06
(7) Externalizing-Antagonism[2] (SCID-II)	0.63	0.85	0 – 4	1.35	1.42							1.	.13	−.02	.32
(8) Externalizing-Disinhibition[2] (SCID-II)	0.37	0.65	0 – 3	1.67	2.07								1	−.07	.06
(9) Detachment[2] (SCID-II)	0.40	0.65	0 – 3	1.51	1.51									1	.25
(10) Thought Disorder[2] (SCID-II)	0.15	0.42	0 – 3	2.99	9.43										1

Note. Correlations were calculated by Pearson's formula. [1] = Standardized by populations norms. [2] = Diagnosis counts.

Table S4

Descriptive Statistics and Bivariate Correlations of Scale Scores in Sample 4 (N=712)

	M	*SD*	Skew	Kurtosis	Bivariate Correlations										
					**1**	**2**	**3**	**4**	**5**	**6**	**7**	**8**	**9**	**10**	**11**
(1) General Interpersonal Distress (CSIP-64)	44.67	25.58	0.66	0.17	1	.48	−.33	−.26	−.40	−.06	.52	.31	.38	.46	.33
(2) Neuroticism (BFI-2)	35.96	10.11	0.10	−0.58		1	−.27	−.26	−.34	.02	.70	.09	.26	.33	.15
(3) Agreeableness (BFI-2)	45.54	7.65	−0.41	−0.21			1	.28	.11	.14	−.24	−.53	−.34	−.44	−.35
(4) Conscientiousness (BFI-2)	43.97	8.29	−0.18	−0.53				1	.27	.13	−.19	−.25	−.61	−.26	−.27
(5) Extraversion (BFI-2)	40.04	8.94	−0.12	−0.48					1	.19	−.20	.09	−.12	−.44	−.09
(6) Openness to Experience (BFI-2)	44.08	7.93	−0.21	−0.53						1	.03	−.01	−.04	−.08	.17
(7) Negative Affect (PID-5-100)	13.12	7.49	0.40	−0.42							1	.30	.42	.30	.35
(8) Antagonism (PID-5-100)	6.69	6.29	1.03	0.48								1	.49	.37	.55
(9) Disinhibition (PID-5-100)	9.83	6.09	0.47	−0.22									1	.40	.51
(10) Detachment (PID-5-100)	7.14	5.45	0.75	0.29										1	.48
(11) Psychoticism (PID-5-100)	7.26	5.96	0.87	0.29											1

Note. Correlations were calculated by Pearson’s formula. Descriptive statistics were calculated for unstandardized scale scores.

Table S5

Estimated Parameters of Evaluated and Considered Candidate Models

			Latent Factors				Latent Classes			Observed Variables	
Model	Abbreviation	Application in Sample	Factor	Factor Means	Factor Variances	Theoretical Factor Distribution	Latent Classes	Determining the Number of Classes (Criteria)	Class Membership Probabilities	Indicator Intercepts	Indicator Error Variances
Confirmatory Factor Analysis (Perfect Circumplex)	CFA-PC	1-4	3	fixed to 0	fixed to 1	normal	none	n.a.	n.a.	freely estimated	freely estimated
Confirmatory Factor Analysis (Quasi Circumplex)	CFA-QC	1-4	3	fixed to 0	fixed to 1	normal	none	n.a.	n.a.	freely estimated	freely estimated
t-Confirmatory Factor Analysis	*t*-CFA	3,4	3	fixed to 0	fixed to 1	*t*-distribution (as defined by *t* degrees of freedom)	none	n.a.	n.a.	freely estimated	freely estimated
Skew-Confirmatory Factor Analysis	Skew-CFA	not applied	3	fixed to 0	fixed to 1	skew-normal distribution (as defined by skew)	none	n.a.	n.a.	freely estimated	freely estimated
Skew-*t*-Confirmatory Factor Analysis	Skew-*t*-CFA	1	3	fixed to 0	fixed to 1	skew-*t* (as defined by *t* degrees of freedom and skew)	none	n.a.	n.a.	freely estimated	freely estimated
2-Class *t*-Confirmatory Factor Analysis	2-Class-*t*-CFA	mentioned in footnote 3	3	fixed to 0 for the reference class, freely estimated for the second class	fixed to 1	non-normal, consisting of two *t*-distributed latent classes	2	confirmatory	2	freely estimated, fixed between classes	freely estimated, fixed between classes
Semi-Parametric Factor Analysis	SP-FA	1-4	3	fixed to 0 for the reference class, freely estimated for *k*-1 classes	fixed to 1	non-normal, consisting of *k* normally distributed classes that are characterized by a class-specific pattern on the factor means	*k*	exploratory (AICc, BIC)	*k*	freely estimated, fixed between classes	freely estimated, fixed between classes
Non-Parametric Factor Analysis	NP-FA	not applied because model is not identified	3	fixed to 0 for the reference class, freely estimated for *k*-1 classes	fixed to 0	non-normal, consisting of *k* discretely distributed subpopulations that are characterized by a class-specific pattern on the factor means	*k*	exploratory	*k*	freely estimated, fixed between classes	freely estimated, fixed between classes
Latent Class Analysis	LCA	1-4	n.a.	n.a.	n.a.	n.a.	*k*	exploratory (AICc, BIC, VLMR, BLRT)	*k*	freely estimated for *k* classes	freely estimated, fixed between classes

Note. n.a. = Not applicable.

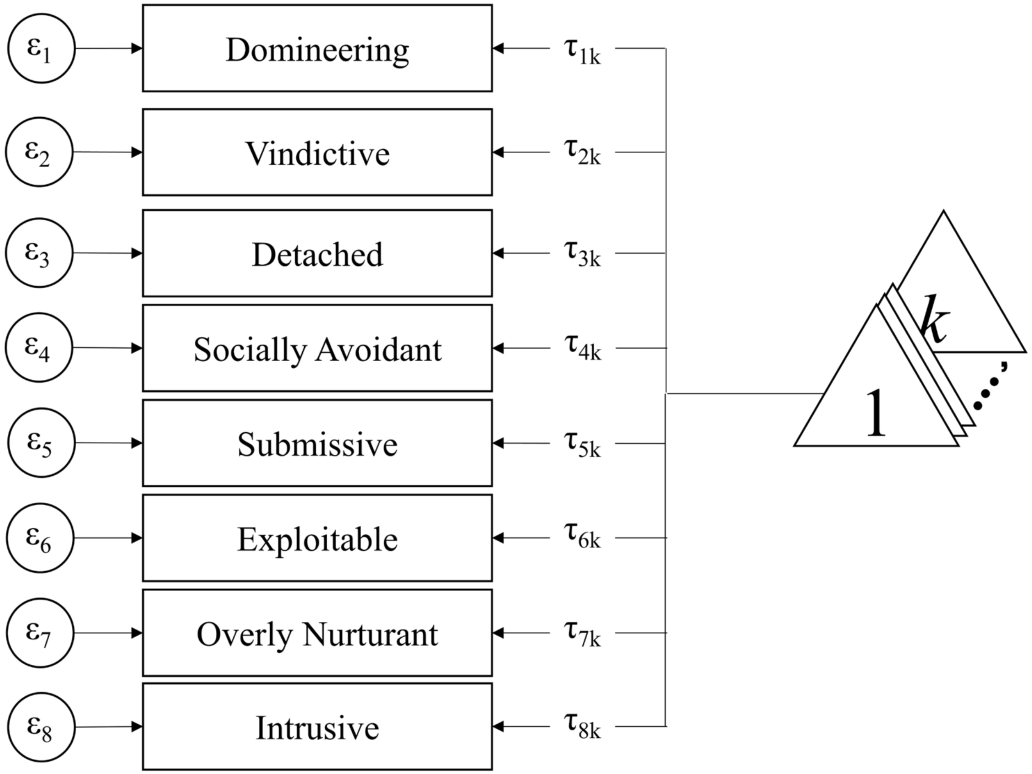

Figure S6. Structural Notation for the LCA model. *Note.* Estimated parameters include error variances (i.e., ε_1-ε_8), class-specific indicator intercepts (τ_{1k}- τ_{8k}), and class membership probabilities. Error variances are invariant over classes.

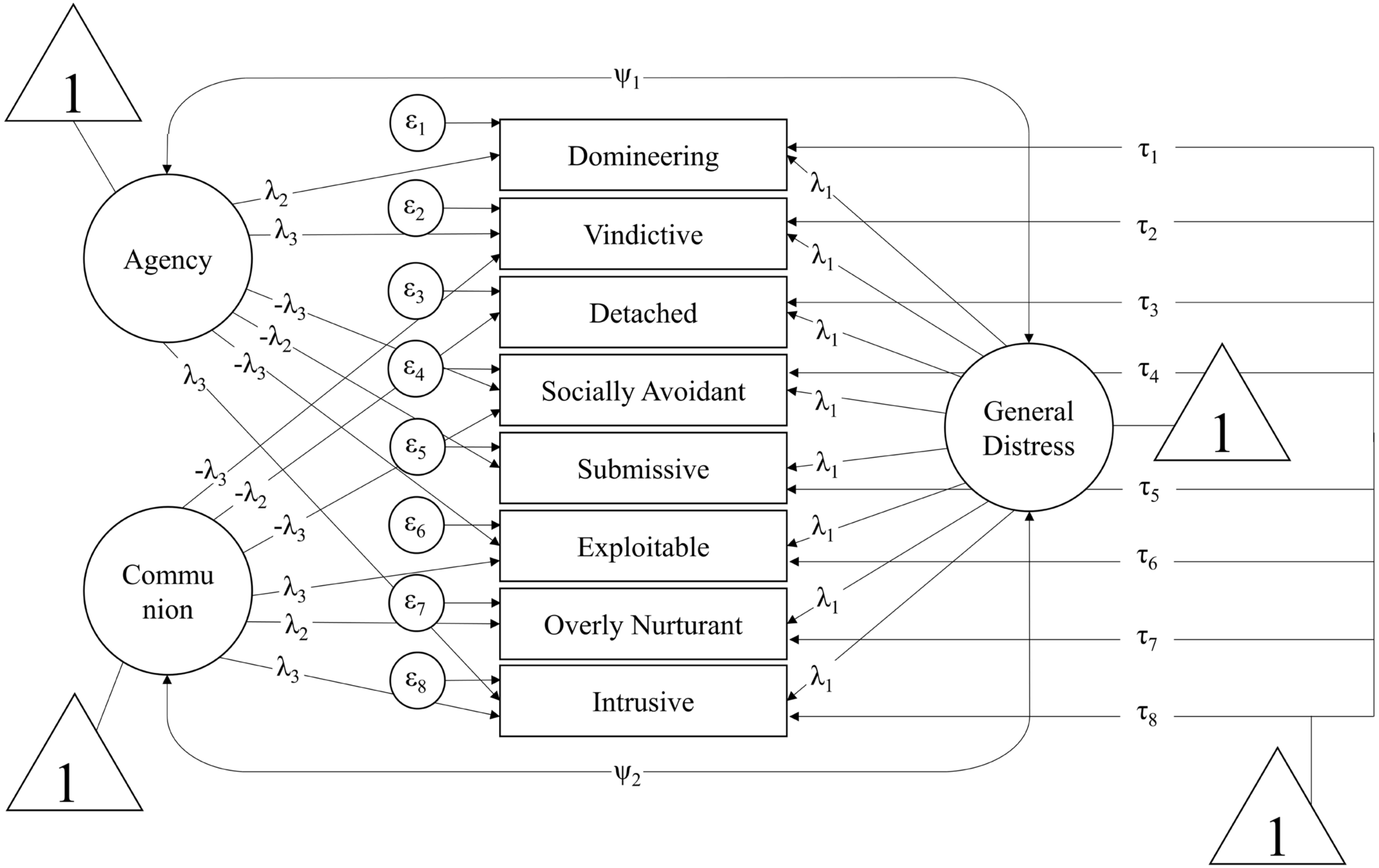

Figure S7. Structural Notation for the CFA-PC model. *Note.* Estimated parameters include two factor loadings (i.e., λ_1, λ_2), eight error variances (i.e., ε_1-ε_8), eight indicator intercepts (τ_1- τ_8) and two factor correlations (ψ_1 and ψ_1). Factor variances are fixed to 1. Subscript numbers indicate equal factor loadings. The ‘equal spacings‘ condition defines factor loadings as $\lambda_3 = \lambda_2 * 0.71$. Factor means are set to 0.

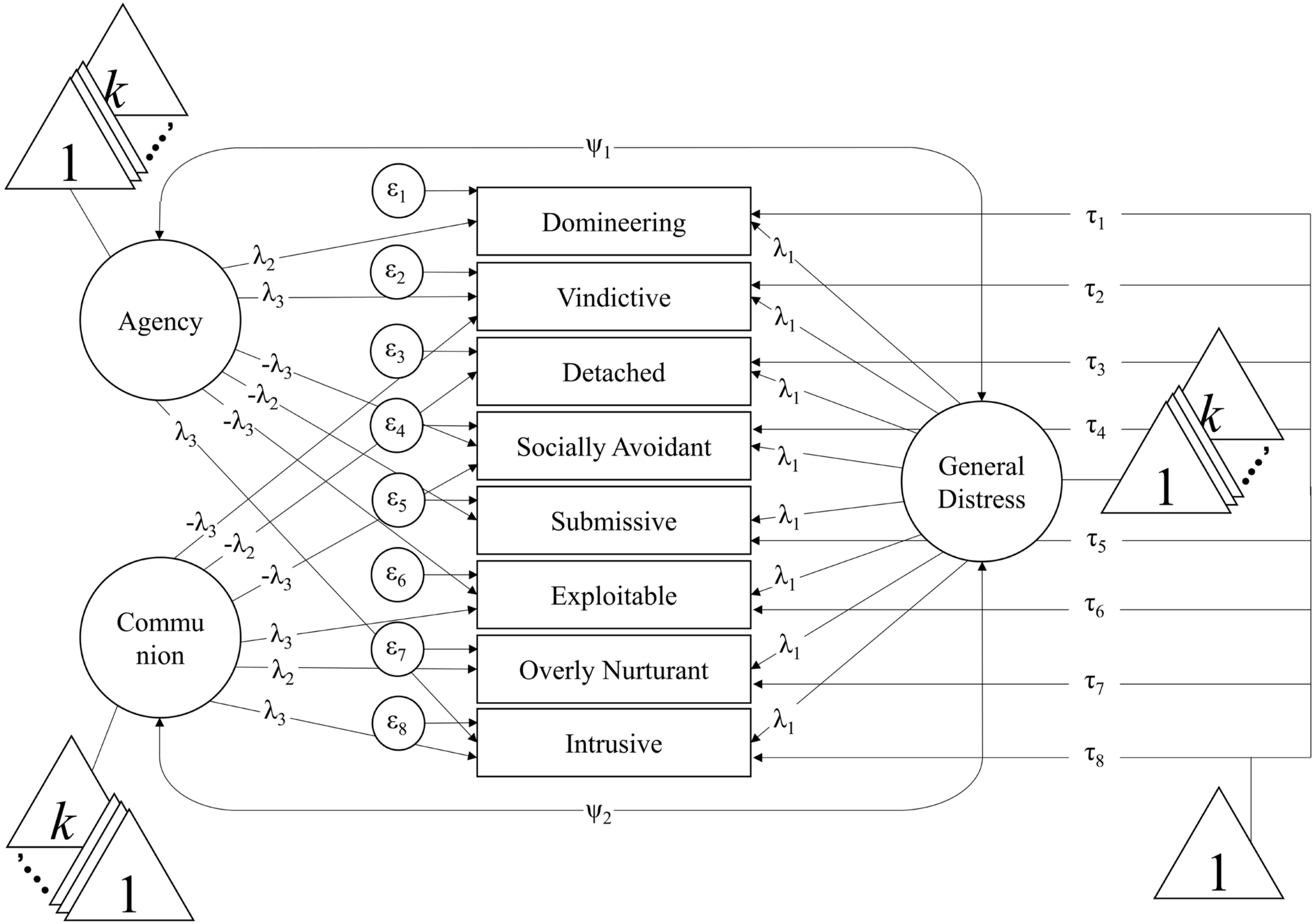

Figure S8. Structural Notation for the SP-FA model. *Note.* Estimated parameters include two factor loadings (i.e., λ_1, λ_2), eight error variances (i.e., ε_1-ε_8), eight indicator intercepts (not displayed; τ_1- τ_8), two factor correlations (ψ_1 and ψ_1), *k*-1 times three factor means, and class membership probabilities. Factor variances are fixed to 1. Subscript numbers indicate equal factor loadings. The 'equal spacings' condition defines factor loadings as $\lambda_3 = \lambda_2 * 0.71$. Factor loadings and indicator intercepts are invariant over classes. The first latent class is defined as the reference group with factor means of 0.

Table S9

Fit Statistics and Likelihood Ratio-Tests of LPA Models to Determine the Optimal Number of Latent Classes in Sample 1 (N = 5400)

						Likelihood-Ratio Test *p*-Value	
Classes	κ	Entropy	Smallest Class Proportion	AIC_C	BIC	VLMR	BLRT
2	25	.844	.422	126953	127118	< .001	/
3	34	.811	.270	123414	123637	< .001	/
4	43	.832	.185	120368	120651	.004	/
5	52	.828	.108	118573	118915	**.046**	/
6	61	.834	.092	117378	117779	.206	< .001
7	70	.821	.084	116383	116842	/	< .001
8	79	.826	.066	115459	115978	/	< .001
9	88	.826	.050	**114771**	**115348**	/	**< .001**
10	97	.827	.030	114207	114843	/	< .001
11	106	.826	.040	113630	114325	/	< .001
12	115	.823	.036	113168	113921	/	< .001
13	124	.826	.020	112776	113587	/	< .001
14	133	.828	.025	112397	113267	/	< .001
15	142	.825	.024	112076	113005	/	< .001

Note. Criterion-specific favored class solutions are highlighted in bold. Likelihood-ratio tests relate to *k* versus *k*-1 model comparisons. *P*-values smaller than .05 and decreases in AIC_C /BIC by 2 points were considered significant. Solutions that yielded class proportions smaller than 5% of the total sample were not considered. κ = Number of free parameters. AIC_C = corrected Akaike's information criterion. BIC = Bayesian information criterion. BLRT = Bootstrapped likelihood-ratio test. VLMR = Vuong-Lo-Mendel-Rubin test.

Table S10

Fit Statistics and Likelihood-Ratio Tests of LPA Models to Determine the Optimal Number of Latent Classes in Sample 2 (N=491)

						Likelihood-Ratio Test p-Value	
Classes	κ	Entropy	Smallest Class Proportion	AIC_C	BIC	VLMR	BLRT
2	25	.894	.430	13189	13291	< .001	/
3	34	.855	.303	12910	13047	**.005**	/
4	43	.868	.157	12736	12908	.145	< .001
5	52	.867	.112	12601	12806	/	< .001
6	61	.859	.112	12489	12727	/	< .001
7	70	.870	.067	12422	12692	/	< .001
8	79	.880	.075	**12361**	**12662**	/	**< .001**
9	88	.890	.033	12315	12646	/	< .001
10	97	.894	.032	12286	12645	/	< .001
11	106	.891	.033	12259	12645	/	< .001
12	115	.892	.018	12230	12641	/	< .001
13	124	.895	.018	12274	12710	/	< .001
14	133	.900	.018	12189	12647	/	< .001
15	142	.908	.014	12166	12645	/	< .001

Note. Criterion-specific favored class solutions are highlighted in bold. Likelihood-ratio tests relate to k versus k-1 model comparisons. P-values smaller than .05 and decreases in AIC_C /BIC by 2 points were considered significant. Solutions that yielded class proportions smaller than 5% of the total sample were not considered. κ = Number of free parameters. AIC_C = corrected Akaike's information criterion. BIC = Bayesian information criterion. BLRT = Bootstrapped likelihood-ratio test. VLMR = Vuong-Lo-Mendel-Rubin test.

Table S11

Fit Statistics and Likelihood-Ratio Tests of Hybrid Models to Determine the Optimal Number of Latent Classes in Sample 3 (N=656)

						Likelihood-Ratio Test *p*-Value	
Classes	κ	Entropy	Smallest Class Proportion	AIC_C	BIC	VLMR	BLRT
2	25	.854	.471	15434	15544	< .001	/
3	34	.853	.290	15024	15173	**< .001**	/
4	43	.838	.130	14767	14954	.524	< .001
5	52	.833	.084	14587	14811	/	< .001
6	61	.835	.092	**14446**	**14707**	/	**< .001**
7	70	.850	.011	14301	14598	/	< .001
8	79	.852	.011	14189	14522	/	< .001
9	88	.856	.011	14098	14465	/	< .001
10	97	.856	.011	14028	14429	/	< .001
11	106	.861	.011	13958	14392	/	< .001
12	115	.863	.011	13909	14376	/	< .001
13	124	.859	.012	13867	14365	/	< .001
14	133	.867	.010	13818	14346	/	< .001
15	142			Non-Convergence			

Note. Criterion-specific favored class solutions are highlighted in bold. Likelihood-ratio tests relate to *k* versus *k*-1 model comparisons. *P*-values smaller than .05 and decreases in AIC_C /BIC by 2 points were considered significant. Solutions that yielded class proportions smaller than 5% of the total sample were not considered. κ = Number of free parameters. AIC_C = corrected Akaike's information criterion. BIC = Bayesian information criterion. BLRT = Bootstrapped likelihood-ratio test. VLMR = Vuong-Lo-Mendel-Rubin test.

Table S12

Fit Statistics and Likelihood-Ratio Tests of LPA Models to Determine the Optimal Number of Latent Classes in Sample 4 (N=712)

						Likelihood-Ratio Test p-Value	
Classes	κ	Entropy	Smallest Class Proportion	AIC_C	BIC	VLMR	BLRT
2	25	0.879	.326	31968	32080	**< .001**	/
3	34	0.910	.104	31384	31535	.055	< .001
4	43	0.859	.093	**31089**	**31279**	/	**< .001**
5	52	0.878	.034	30796	31025	/	< .001
6	61	0.872	.035	30616	30883	/	< .001
7	70	0.879	.029	30461	30765	/	< .001
8	79						
9	88						
10	97						
11	106			Non-Convergence			
12	115						
13	124						
14	133						
15	142						

Note. Criterion-specific favored class solutions are highlighted in bold. Likelihood-ratio tests relate to k versus k-1 model comparisons. P-values smaller than .05 and decreases in AIC_C /BIC by 2 points were considered significant. Solutions that yielded class proportions smaller than 5% of the total sample were not considered. κ = Number of free parameters. AIC_C = corrected Akaike's information criterion. BIC = Bayesian information criterion. BLRT = Bootstrapped-likelihood-ratio test. VLMR = Vuong-Lo-Mendel-Rubin test.

Table S13

Fit Statistics for Semi-Parametric Factor Analysis Models (SP-FA) to Determine the Optimal Number of Latent Classes for a Hybrid Model in Sample 1 (N = 5400)

Classes	κ	Entropy	AIC_C	BIC	Smallest Class Proportion
2	24	.708	110877	111035	.177
3	28	.666	110754	110938	.082
4	32	.525	110611	110822	.140
5	36	.582	**110438**	**110674**	.088
6	40	.591	110360	110624	.041
7	44	.630	110310	110599	.011
8	48	.638	110261	110576	.007
9	52	.631	110223	110564	.005

Note. Criterion-specific favored class solutions are highlighted in bold. Smallest class proportions were based on the most likely latent class membership. Solutions that yielded class proportions smaller than 5% of the total sample were not considered. Decreases in AIC_C /BIC by 2 points were considered significant. κ = Number of free parameters. AIC_C = corrected Akaike's information criterion. BIC = Bayesian information criterion.

Table S14

Fit Statistics for Semi-Parametric Factor Analysis Models (SP-FA) to Determine the Optimal Number of Latent Classes for a Hybrid Model in Sample 2 (N=491)

Classes	κ	Entropy	AIC_C	BIC	Smallest Class Proportion
2	24	.730	12395	12493	.424
3	28	.704	12338	12452	.281
4	32	.738	**12305**	**12435**	.090
5	36				
6	40				
7	44		Non-Convergence		
8	48				
9	52				

Note. Criterion-specific favored class solutions are highlighted in bold. Smallest class proportions were based on the most likely latent class membership. Solutions that yielded class proportions smaller than 5% of the total sample were not considered. Decreases in AIC_C /BIC by 2 points were considered significant. κ = Number of free parameters. AIC_C = corrected Akaike's information criterion. BIC = Bayesian information criterion.

Table S15

Fit Statistics for Semi-Parametric Factor Analysis Models (SP-FA) to Determine the Optimal Number of Latent Classes for a Hybrid Model in Sample 3 (N=656)

Classes	κ	Entropy	AIC_C	BIC	Smallest Class Proportion
2	24	.752	**13575**	**13681**	.095
3	28	.762	13542	13665	.022
4	32	.690	13518	13658	.011
5	36	.700	13507	13664	.009
6	40	.722	13500	13674	.009
7	44	.720	13490	13680	.009
8	48	.748	13484	13692	.009
9	52	.764	13483	13707	.009

Note. Criterion-specific favored class solutions are highlighted in bold. Smallest class proportions were based on the most likely latent class membership. Solutions that yielded class proportions smaller than 5% of the total sample were not considered. Decreases in AIC_C /BIC by 2 points were considered significant. κ = Number of free parameters. AIC_C = corrected Akaike's information criterion. BIC = Bayesian information criterion.

Table S16

Fit Statistics for Semi-Parametric Factor Analysis Models (SP-FA) to Determine the Optimal Number of Latent Classes for a Hybrid Model in Sample 4 (N=712)

Classes	κ	Entropy	AIC_C	BIC	Smallest Class Proportion
2	24	.928	30033	30141	.052
3	28	.833	**29908**	**30034**	.081
4	32	.862	29850	29993	.020
5	36				
6	40				
7	44	Non-Convergence			
8	48				
9	52				

Note. Criterion-specific favored class solutions are highlighted in bold. Smallest class proportions were based on the most likely latent class membership. Solutions that yielded class proportions smaller than .05 were not considered. Decreases in AIC_C /BIC by 2 points were considered significant. κ = Number of free parameters. AIC_C = corrected Akaike's information criterion. BIC = Bayesian information criterion.
