# Supplementary material for: The Latent Structure of Interpersonal Problems: Validity of Dimensional, Categorical, and Hybrid Models
Source: J Abnorm Psychol. 2019 Sep 26;128(8):823–39. doi: 10.1037/abn0000460 (PMC6816327; doi:10.1037/abn0000460)
Supplement: Supplementary file 1 [file ABN-2018-0746_Suppl.zip › latent structure of IP _min_rev_ online supplement b.pdf]

Table S17

Fit Statistics and Variance Explained in External Variables of Candidate Models for the German Translation of the IIP-64 in Sample 1 (N = 5400)

						R^2									
						SCL-90-R									
	Model	κ	LL	AIC_C	BIC	GSI	SO	OC	IS	DE	AN	HO	PA	PI	PS
Dimensional	3-Factor CFA-PC	20	−55686	111545	111413	**.42**	**.11**	**.31**	**.47**	**.33**	**.21**	**.28**	**.14**	**.38**	**.35**
	3-Factor CFA-QC	23	−55649	111496	111344	**.42**	**.11**	**.31**	**.47**	**.33**	**.21**	**.28**	**.14**	.37	**.35**
	3-Factor skew-*t*-CFA	24	−55144	**110337**	**110495**	**.42**	**.11**	**.31**	**.47**	**.33**	**.21**	**.28**	**.14**	**.38**	**.35**
Hy.	5-Class SP-FA[a,b]	36	−55183	110438	110674	**.42**	**.11**	**.31**	**.47**	**.33**	**.21**	**.28**	**.14**	**.38**	**.35**
Categorical	5-Class LCA[c]	52	−59234	118573	118915	.35	.09	.26	.40	.28	.17	.16	.12	.29	.28
	9-Class LCA[a,b,d]	88	−57296	114771	115348	.36	.09	.26	.42	.29	.18	.20	.13	.32	.29

Note. Multiple linear regressions were based on dimensional and categorical latent scores. Most favorable values are highlighted in bold print. R^2 = Variance explained in external variables (adjusted coefficient of determination). κ = Number of free parameters. LL = Log-likelihood. AIC_C = corrected Akaike's information criterion. BIC = Bayesian information criterion. SCL-90-R = Symptom-Checklist-90-Revised. GSI = General Severity Index. SO = Somatization. OC = Obsessive-Compulsive. IS = Interpersonal Sensitivity. DE = Depression. AN = Anxiety. HO = Hostility. PA = Phobic Anxiety. PI = Paranoid Ideation. PS = Psychoticism. [a] = Optimal number of classes via AIC_C. [b] = Optimal number of classes via BIC. [c] = Optimal number of classes via VLMR. [d] = Optimal number of classes via BLRT. Hy. = Hybrid.

Table S18

Fit Statistics and Variance Explained in External Variables of Candidate Models for the IIP-32. Results from Sample 2 (N = 491)

						R^2															
						BSI										BIS	EQ	OAS	DERS	SPQ	PCL
	Model	κ	LL	AIC_C	BIC	GSI	SO	OC	IS	DE	AN	HO	PA	PI	PS	AI					
Dimensional	3-Factor CFA-PC	20	−6210	12545	12463	**.53**	**.29**	**.40**	**.54**	**.45**	**.42**	.36	**.43**	**.46**	**.50**	**.38**	**.23**	**.56**	**.54**	**.54**	**.46**
	3-Factor CFA-QC	23	−6202	12547	12453	**.53**	**.29**	**.40**	.53	**.45**	.41	.36	**.43**	**.46**	**.50**	**.38**	**.23**	.55	**.54**	**.54**	**.46**
	3-Factor *t*-CFA	21	−6187	12418	12505	**.53**	**.29**	**.40**	**.54**	**.45**	**.42**	.36	**.43**	**.46**	**.50**	**.38**	**.23**	**.56**	**.54**	**.54**	**.46**
Hy.	4-Class SP-FA[a,b]	32	−6118	**12305**	**12435**	.52	**.29**	**.40**	.53	.44	**.42**	.35	.42	**.46**	.48	.37	.22	.55	.53	.53	.45
Categorical	3-Class LPA[c]	34	−6418	12910	13047	.39	.20	.32	.44	.34	.31	.15	.34	.29	.35	.30	.03	.45	.42	.38	.31
	8-Class LPA[a,b,d]	79	−6086	12361	12662	.46	.24	.35	.51	.41	.35	.26	.36	.37	.43	.33	.12	.51	.50	.47	.40

Note. Multiple linear regressions were based on dimensional and categorical latent scores. Most favorable values are highlighted in bold print. κ = Number of free parameters. LL = Log-likelihood. AIC_C = corrected Akaike's information criterion. R^2 = Variance explained in external variables (adjusted coefficient of determination). BIC = Bayesian information criterion. BSI = Brief Symptom Inventory. GSI = General Severity Index. SO = Somatization. OC = Obsessive-Compulsiveness. IS = Interpersonal Sensitivity. DE = Depression. AN = Anxiety. HO = Hostility. PA = Phobic Anxiety. PI = Paranoid Ideation. PS = Psychoticism. BIS = Baratt Impulsiveness Scale. AI = Attentional impulsiveness. EQ = Empathy Quotient. OAS = The Other as Shamer Scale. DERS = Difficulties in Emotion Regulation Scale. SPQ = Schizotypal Personality Scale. PCL = Posttraumatic Stress Disorder Checklist Scale. [a] = Optimal number of classes via AIC_C. [b] = Optimal number of classes via BIC. [c] = Optimal number of classes via BIC. Hy. = Hybrid.

Table S19

Fit Statistics and Variance Explained in External Variables of Candidate Models for the IIP-64 in Sample 3 (N = 656)

						R^2								
						Diagnosis Counts								
	Model	κ	LL	AIC_C	BIC	TOT	Axis-I	Axis-II	I-FEAR	I-DIST	E-ANT	E-DIS	DET	THO
Dimensional	3-Factor CFA-PC	20	−6790	13622	13710	**.18**	**.09**	**.14**	**.08**	**.13**	**.13**	**.07**	**.20**	**.03**
	3-Factor CFA-QC	23	−6785	13719	**13617**	**.18**	**.09**	**.14**	**.08**	**.13**	**.13**	**.07**	**.20**	**.03**
	3-Factor *t*-CFA	21	−6741	**13525**	**13617**	**.18**	**.09**	**.14**	**.08**	**.13**	**.13**	**.07**	**.20**	**.03**
H.	2-Class SP-FA[a,b]	24	−6763	13575	13681	**.18**	**.09**	**.14**	**.08**	**.13**	**.13**	**.07**	**.20**	**.03**
Categorical	3-Class LCA[c]	34	−7476	15024	15173	.12	.07	.07	.06	.10	.03	.00	.10	.00
	6-Class LCA[a,b,d]	61	−7156	14446	14707	.15	.08	.10	.07	.12	.07	.02	.15	.00

Note. Multiple linear regressions were based on dimensional and categorical latent scores. Most favorable values are highlighted in bold print. R^2 = Variance explained in external variables (adjusted coefficient of determination). κ = Number of free parameters. LL = Log-likelihood. AIC_C = corrected Akaike's information criterion.
BIC = Bayesian information criterion. TOT = Total diagnoses. Axis-I+II = Axis-I+II diagnoses. I-FEAR = Internalizing-fear diagnoses. I-DIST = Internalizing-distress diagnoses. E-ANT = Externalizing-antagonism diagnoses. E-DIS = Externalizing-disinhibition diagnoses. DET = Detachment diagnoses. THO = Thought disorder diagnoses. [a] = Optimal number of classes via AIC_C. [b] = Optimal number of classes via BIC. [c] = Optimal number of classes via VLMR. [d] = Optimal number of classes via BLRT. H. = Hybrid.

Table S20

Fit Statistics and Variance Explained in External Variables of Candidate Models for the CSIP in Sample 4 (N = 712)

	Model	κ	LL	AIC_C	BIC	R^2									
						BFI-2					PID-5-100				
						O	C	E	A	N	PSY	DIS	DET	ANT	NEG
Dimensional	3-Factor CFA-PC	20	−15070	30271	30181	.00	**.07**	**.56**	**.38**	**.23**	.14	**.16**	**.33**	**.29**	**.27**
	3-Factor CFA-QC	23	−15056	30262	30159	.00	**.07**	.55	.37	**.23**	.14	.15	**.33**	**.29**	**.27**
	3-Factor *t*-CFA	21	−14894	**29831**	**29926**	.00	**.07**	.55	**.38**	.22	.14	.15	**.33**	.28	**.27**
H.	3-Class SP-FA[a,b]	28	−14925	29908	30034	.00	**.07**	.55	.37	.22	**.15**	**.16**	.32	**.29**	**.27**
Categorical	2-Class LCA[c]	25	−15958	31968	32080	.00	.05	.12	.05	.15	.06	.09	.12	.06	.19
	4-Class LCA[a,b,d]	43	−15498	31089	31279	.00	.04	.23	.16	.20	.06	.09	.18	.13	.20

Note. Multiple linear regressions were based on dimensional and categorical factor scores. Most favorable values are highlighted in bold print. R^2 = Variance explained in external variables (adjusted coefficient of determination). κ = Parameter count. LL = Log-likelihood. AIC_C = corrected Akaike's information criterion. BIC = Bayesian information criterion. BFI-2 = Big-Five-Inventory 2. PID-5-100 = Personality Inventory for DSM-5. O = Openness to Experience. C = Conscientiousness. E = Extraversion. A = Agreeableness. N = Neuroticism. PSY = Psychoticism. DIS = Disinhibition. DET = Detachment. ANT = Antagonism. NEG = Negative Affectivity. [a] = Optimal number of classes via AIC_C. [b] = Optimal number of classes via BIC. [c] = Optimal number of classes via VLMR. [d] = Optimal number of classes via BLRT. H. = Hybrid models.

Estimated Factor Score Distributions for Interpersonal Dimensions in Sample 1

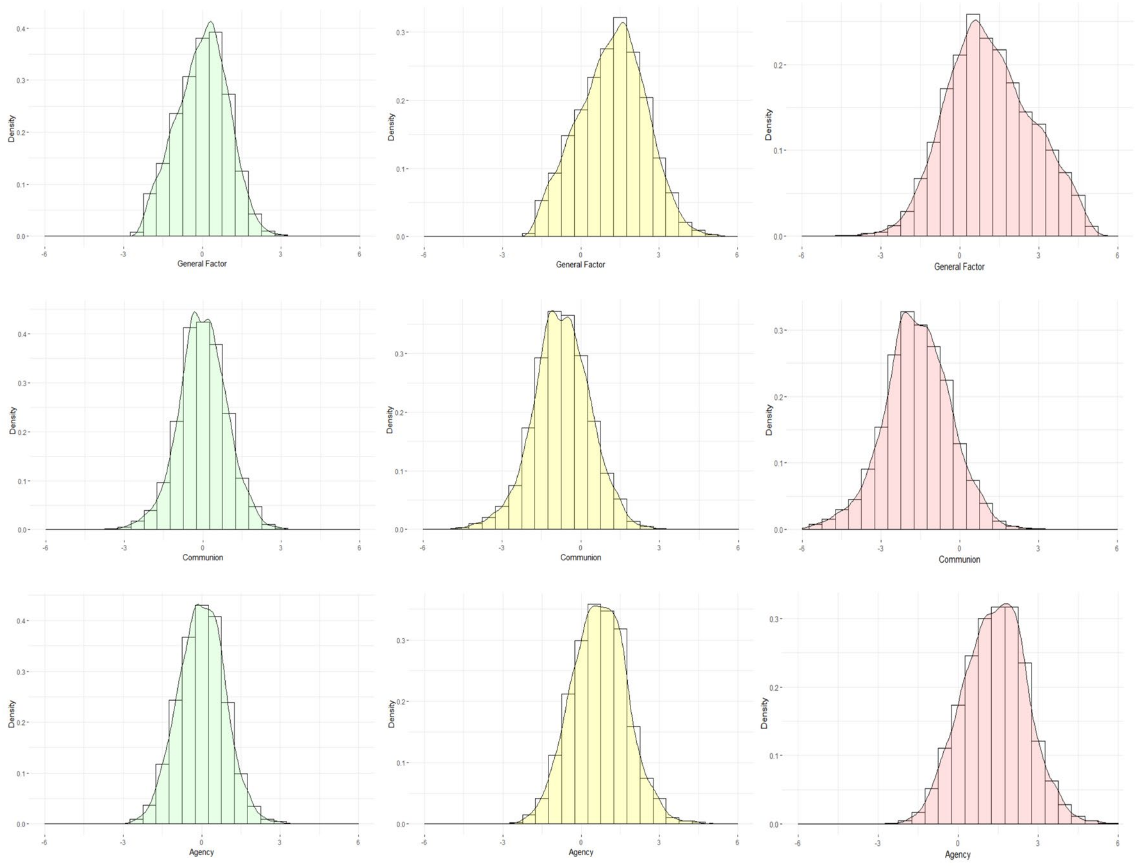

Figure S21. CFA (in green) assumes a single latent normal distribution. Skew-t-CFA (in yellow) assumes a single latent non-normal skewed *t*-distribution. 5-Class-SP-FA (in red) assumes that the latent factor distribution consists of nine normally distributed latent subpopulations. Predicted factor scores between models have correlations greater than $r >$ .99.

Estimated Factor Score Distributions for Interpersonal Dimensions in Sample 2

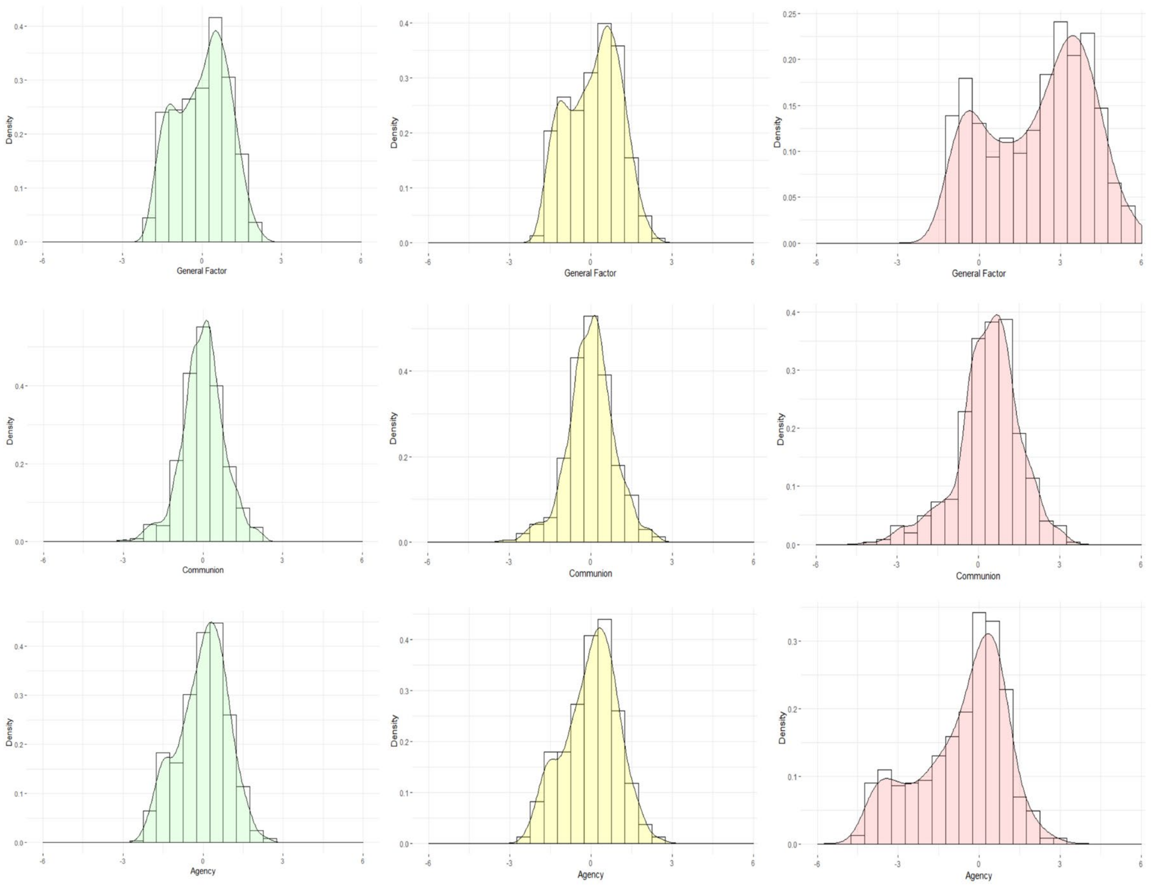

Figure S22. CFA (in green) assumes a single latent normal distribution for each dimension. *t*-CFA (in yellow) assumes that the latent factor distribution consists of two *t*-distributed latent subpopulations. 4-Class-SP-FA (in red) assumes that the latent factor distribution consists of four normally distributed latent subpopulations. Predicted factor scores between models have correlations greater than $r > .94$.

Estimated Factor Score Distributions for Interpersonal Dimensions in Sample 3

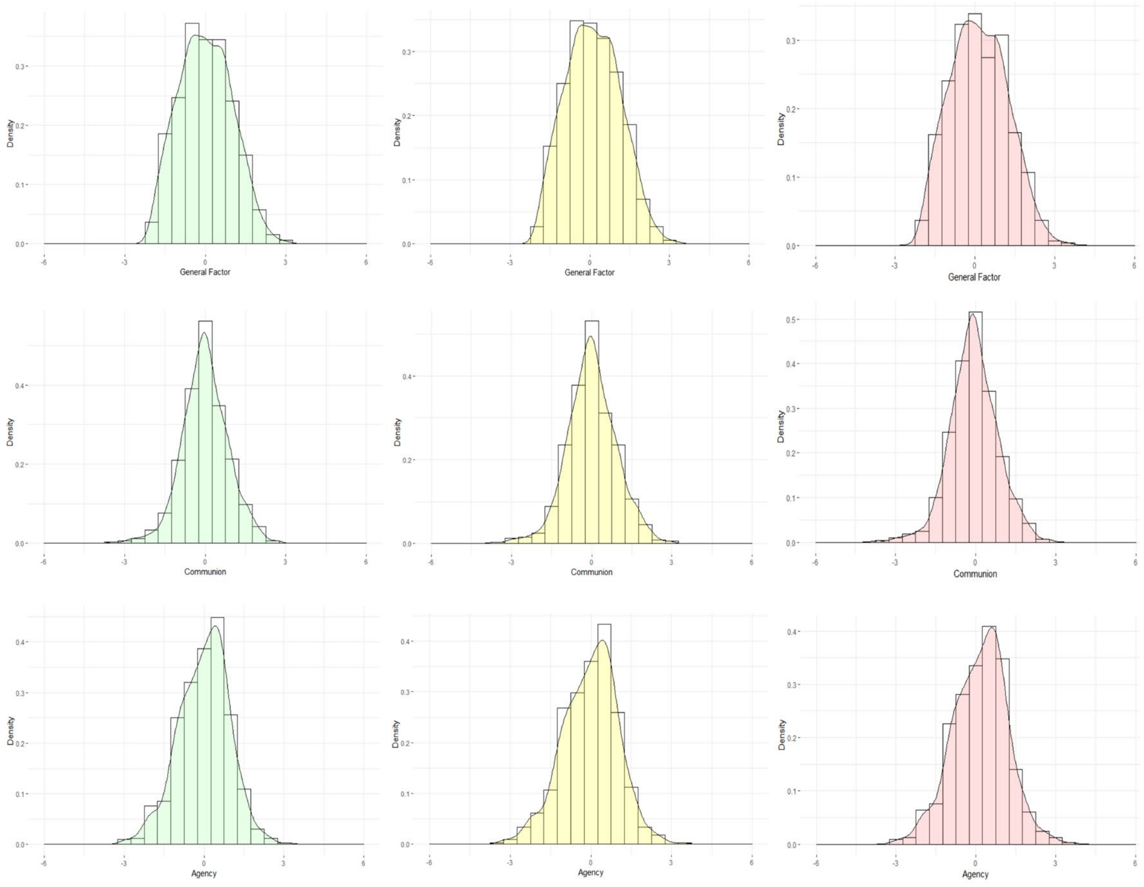

Figure S23. CFA (in green) assumes a single latent normal distribution. *t*-CFA (in yellow) assumes a single latent non-normal *t*-distribution. 2-Class-SP-FA (in red) assumes that the latent factor distribution consists of eight normally distributed latent subpopulations. Predicted factor scores between models have correlations greater than $r > .98$.

Estimated Factor Score Distributions for Interpersonal Dimensions in Sample 4

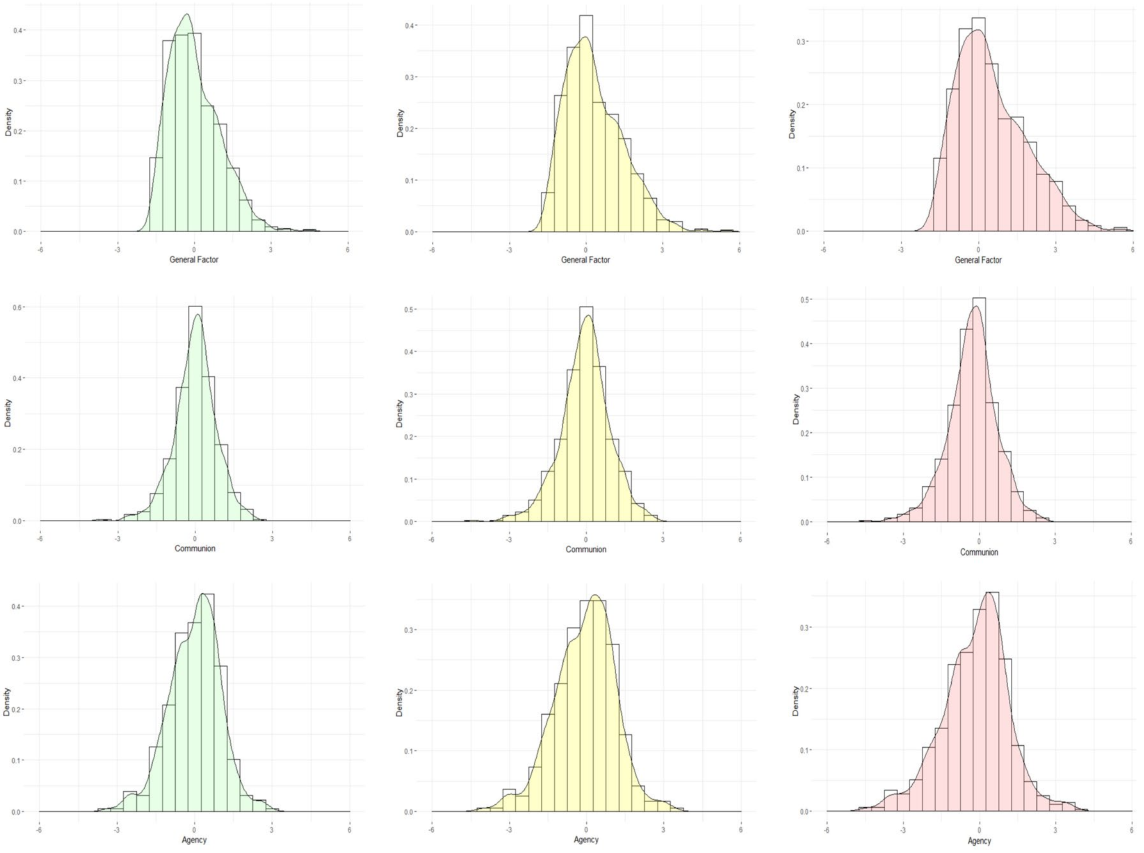

Figure S24. CFA (in green) assumes a single latent normal distribution. *t*-CFA (in yellow) assumes a single latent non-normal *t*-distribution. 3-Class-SP-FA (in red) assumes that the latent factor distribution consists of four normally distributed latent subpopulations. Predicted factor scores between models have correlations greater than $r > .98$.

R Code S25

Lavaan Model Specification for the CFA-PC Model of Interpersonal Problems with Equal Communalities and Equal Spacings. The General Factor Loads Equally Strong on the Indicators.

```
GF    =~ L1 * pa + L1 * bc + L1 * de + L1 * fg + L1 * hi + L1 * jk + L1 * lm + L1 * no
AG    =~ L2 * pa + start(1) * pa + L3 * bc + L3.N * fg + L2.N * hi + L3.N * jk + L3 * no

COM =~ L3.n * bc + L2.n * de + L3.n * fg + L3 * jk + L2 * lm + start(1) * lm + L3 * no

AG ~~ 0 * COM

L2 > 0

L2N == -1 * L2

L3N == -1 * L3

L3 == 0.707 * L2
```

R Code S26

Model Specification for the Skew-t-CFA Model of Interpersonal Problems in MPlus as Accessed by the MplusAutomation Package in R Studio

```
model_cfa_skewt <- mplusObject(
  TITLE = "Skew-t Factor Analysis.",
  ANALYSIS = "Type=general. estimator = MLR. distribution=SKEWT.",
  OUTPUT = "SAMPSTAT. STANDARDIZED.",
  SAVEDATA = 'FILE = "model_cfa_skewt.dat".',
  MODEL = "
  gf  BY
  pa*1
  pa bc de fg hi jk lm no (lg).
  dom BY
  pa*1
  pa (lz)
  bc no (ld)
  fg jk (ldn)
  hi (lzn).
  lov BY
  lm*1
  de (lzn)
  fg bc (ldn)
  jk no (ld)
  lm (lz).
  gf@1.
  dom@1.
  lov@1.
  dom WITH lov @0.
  ",
  MODELCONSTRAINT= "
  lz > 0.
  ldn = -1*ld.
  lzn = -1*lz.
  ld = .707*lz.
  ",
  PLOT = "type = plot3.",
  usevariables = c("pa","bc", "de", "fg","hi", "jk", "lm", "no"),
  rdata = dat)
fit <- mplusModeler(model_cfa_skewt, "mplus_cfa_skewt.dat",
                modelout = "model_cfa_skewt.inp", check = TRUE, run = 1)
```

R Code S27

Model Specification for the SP-FA Model of Interpersonal Problems in MPlus as Accessed by the MplusAutomation Package in R Studio

```
model_spfa3_3 <- mplusObject(
  TITLE = "SP-FA Model;",
  VARIABLE = "classes=c(3);",
  ANALYSIS = "Type=mixture;starts = 4000;
  algorithm = integration; Processors = 8(starts);",
  OUTPUT = "TECH1; TECH8; SAMPSTAT;",
  SAVEDATA = 'FILE = "model_spfa3_3.dat";
  SAVE = CPROBABILITIES;',
  MODEL = " %OVERALL%
  gf  BY
  pa*1
  pa bc de fg hi jk lm no (lg);
  dom BY
  pa*1
  pa (l2)
  bc no (l3)
  fg jk (l3n)
  hi (l2n);
  lov BY
  lm*1
  de (l2n)
  fg bc (l3n)
  jk no (l3)
  lm (l2);
  gf@1;
  dom@1;
  lov@1;
  dom WITH lov @0;

  %c#1%
  [gf@0];
  [dom@0];
  [lov@0];
  dom WITH lov @0;

  %c#2%
  [gf];
  [dom];
  [lov];
  dom WITH lov @0;

  %c#3%
  [gf];
  [dom];
  [lov];
  dom WITH lov @0;
  ",
  MODELCONSTRAINT= "
  l2 > 0 ;
  l3n = -1*l3 ;
  l2n = -1*l2 ;
  l3 = .707*l2;
  ",
  PLOT = "type = plot3;",
  usevariables = c("pa","bc", "de", "fg","hi", "jk", "lm", "no"),
  rdata = dat)
fit <- mplusModeler(model_spfa3_3, "mplus_spfa3_3.dat",
              modelout = "model_spfa3_3.inp", check = TRUE, run = 1)
```
